# Two-Dimensional Transition Metal Dichalcogenide Based Biosensors: From Fundamentals to Healthcare Applications

**DOI:** 10.3390/bios13020169

**Published:** 2023-01-21

**Authors:** Abdul Kaium Mia, M. Meyyappan, P. K. Giri

**Affiliations:** 1 Centre for Nanotechnology, Indian Institute of Technology Guwahati, Guwahati 781039, India; 2 Department of Physics, Indian Institute of Technology Guwahati, Guwahati 781039, India

**Keywords:** biosensors, 2D materials, transition metal dichalcogenides, point of care, electrochemical sensing, optical sensing, electrical sensing

## Abstract

There has been an exponential surge in reports on two-dimensional (2D) materials ever since the discovery of graphene in 2004. Transition metal dichalcogenides (TMDs) are a class of 2D materials where weak van der Waals force binds individual covalently bonded X–M–X layers (where M is the transition metal and X is the chalcogen), making layer-controlled synthesis possible. These individual building blocks (single-layer TMDs) transition from indirect to direct band gaps and have fascinating optical and electronic properties. Layer-dependent opto-electrical properties, along with the existence of finite band gaps, make single-layer TMDs superior to the well-known graphene that paves the way for their applications in many areas. Ultra-fast response, high on/off ratio, planar structure, low operational voltage, wafer scale synthesis capabilities, high surface-to-volume ratio, and compatibility with standard fabrication processes makes TMDs ideal candidates to replace conventional semiconductors, such as silicon, etc., in the new-age electrical, electronic, and opto-electronic devices. Besides, TMDs can be potentially utilized in single molecular sensing for early detection of different biomarkers, gas sensors, photodetector, and catalytic applications. The impact of COVID-19 has given rise to an upsurge in demand for biosensors with real-time detection capabilities. TMDs as active or supporting biosensing elements exhibit potential for real-time detection of single biomarkers and, hence, show promise in the development of point-of-care healthcare devices. In this review, we provide a historical survey of 2D TMD-based biosensors for the detection of bio analytes ranging from bacteria, viruses, and whole cells to molecular biomarkers via optical, electronic, and electrochemical sensing mechanisms. Current approaches and the latest developments in the study of healthcare devices using 2D TMDs are discussed. Additionally, this review presents an overview of the challenges in the area and discusses the future perspective of 2D TMDs in the field of biosensing for healthcare devices.

## 1. Introduction

The discovery of graphene in 2004 set a new benchmark in the two-dimensional (2D) material research and applications [1]. Monolayer graphene was first mechanically exfoliated from graphite flakes and found to have excellent electrical properties. The extraordinary electrical mobility of suspended graphene reported by Bolotin et al. led the scientific community to conduct further studies on graphene for the ultrafast electronics [2]. High carrier mobility in graphene also aids in optical applications, such as photonics and ultrafast photodetection from ultraviolet to terahertz range [3,4,5]. Due to the aforementioned properties, graphene has been an excellent candidate in various applications such as photodetectors, gas sensors, humidity sensors, biosensors, and others [6,7,8,9,10]. The conducting nature of graphene, owing to zero band gap, has fundamental limitations on sensitivity and detection limits in sensing applications [11]. Even though graphene has extremely high electron mobility, the absence of a finite band gap limits its application in field effect transistors (FETs) [12]. Tremendous efforts were made to create a band gap in graphene by chemical doping, AB-stacked bilayer graphene layer, and other approaches, but only a few hundred of meV were achieved [13,14]. The fundamental limitations of graphene and its derivatives led to the search for graphene-like 2D materials with superior properties.

2D materials have the potential for next-generation applications in energy storage, ultrafast electronics, sensing, and others [15,16]. One of the emerging applications of 2D materials lies in the biomedical field, from drug delivery to analyte detection [17,18]. Detection of bioanalytes is a prerequisite for disease diagnosis, progress, and further treatment. Enabled by their high density of surface sites, 2D materials, such as transition metal dichalcogenides (TMDs), hexagonal boron nitride (hBN), Mxenes, and graphite carbon nitride (g-C_3_N_4_), have tremendous potential as transducer elements in different biosensing applications [18,19,20,21]. 2D TMDs with graphene-like planar structure, high fluorescence (FL) emission and quenching, high carrier mobility, high surface-to-volume ratio, and compatibility with modern fabrication technologies are the most suitable alternatives to the graphene [22,23]. In TMDs, weak van der Waals forces bind individual covalently bonded X–M–X layers (where M is the transition metal and X is the chalcogen), making layer-controlled synthesis possible [24]. Unlike graphene, most TMDs have semiconducting electrical properties and have been studied extensively for their application in future integrated electronic circuits [25,26]. Unique optoelectronic properties arise when multilayer TMDs are reduced to monolayers, and the electronic band structure becomes direct from indirect along with strong photoluminescence (PL) and large exciton binding energy [27,28]. TMDs have been recently explored in several applications ranging from photodetection, light emission, bio-imaging, gas sensing, drug delivery, and others [29,30,31,32,33,34].

**Figure 1:**
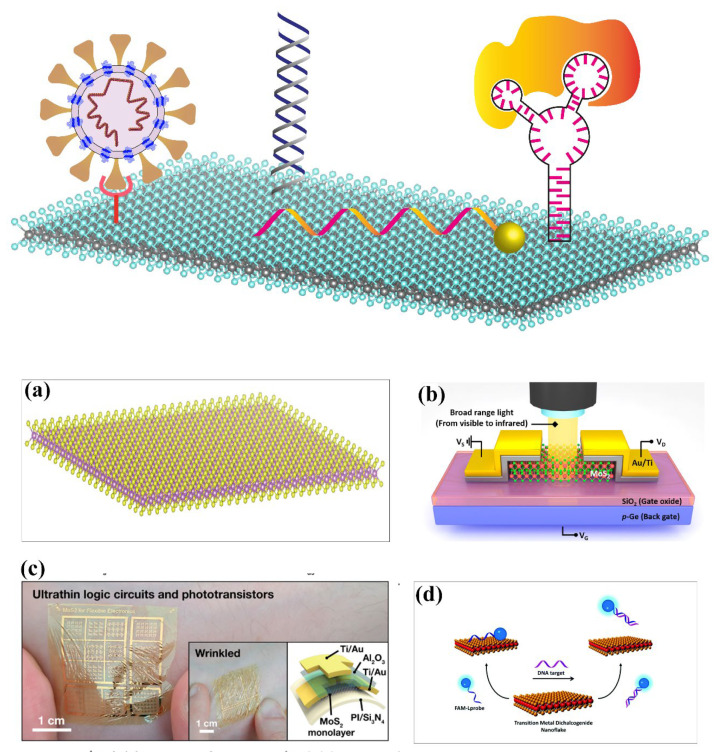
(**a**) Planar structure and high surface-to-volume ratio of 2D TMD; (**b**) 2D TMDs in photodetector applications. Reprinted with permission from ref. [34]. Copyright 2019 American Chemical Society; (**c**) Mechanically flexible properties of TMDs for wearable electronics, reprinted with permission from ref. [44]. Copyrights 2021 American Chemical Society; (**d**) TMDs have efficient charge transfer properties, reprinted with permission from ref. [43]. Copyright 2016 Royal Society of Chemistry.

A finite band gap semiconducting material is crucial for the development of ultrasensitive optical or electronic biosensors [29,35,36]. The excellent properties of TMDs include tunable band gap, planar structure, high carrier mobility, excellent PL emission and quenching, mechanical flexibility, and high on-off ratio, which has driven the development of sensing applications (see Figure 1) [37,38,39,40,41,42,43,44,45]. The atomic layer thickness of TMDs helps the adsorption of bioanalytes into the surface, hence yielding a high sensing response [46]. The layer-dependent tunable band structure, biocompatibility, low toxicity, and properties mentioned above make TMDs suitable candidates as transducers for an ultrasensitive sensor. Selectivity is one of the important parameters for any sensing device that determines its ability to detect an analyte in the presence of similar analytes. With advanced synthesis methods and transfer processes, TMD heterostructures with other 2D materials show improved properties suitable for selective detection. Surface modification of TMDs with the help of defect engineering by various methods, such as plasma treatment, thermal annealing, etc., helps in suitable surface functionalization with specific receptors [47]. Other materials, such as carbon nanotubes (CNT) and silicon nanowires, also exhibit a similar kind of flexibility, but device fabrication using 1D materials and proper alignment are major challenges [48,49,50]. In contrast, the wafer scale synthesis capabilities of TMDs and their planar structure make them compatible with modern-day fabrication technologies [39,51,52]. Another interesting aspect is that they are mechanically flexible and hence, have the potential to be integrated with next-generation flexible wearable point-of-care (PoC) devices [40,53,54,55]. The increase in the active surface site density increases the probability of binding of analytes to the sites of the sensing transducer elements and hence, causing the modulation of opto-electronic properties, which results in highly sensitive biosensors with improved detection limit [36,56,57]. The surface area per gram (SAPG) of TMDs increases substantially when reduced to a few layers from bulk. MoS_2_ shows a SAPG value increase from 8.4 to 25 m^2^ per gram [58]. The higher value of SAPG and low electrical noise will promote lower detection limits. Among all the TMDs, MoS_2_, MoSe_2_, WS_2_, and WSe_2_ have been studied extensively in terms of synthesis and various applications through fundamental properties. The potential application of other TMD families of materials, such as MoTe_2_ and WTe_2_, is yet to be explored.

As pointed out above, the attractive properties of TMD have led to the exploration of future-generation biosensing applications. Depending upon the working principle, the biosensors can be grouped into three categories: (a) Electrical, (b) Electrochemical, and (c) Optical. The detection mechanism of electrical biosensors is simple and end-user-friendly and detects the bioanalytes in real-time. The effect of binding analytes on TMDs surface via receptor is analogous to the applied gate bias in FETs. The electrical detection method transforms the analyte information in the form of current changes [59,60]. There are two working principles for electrochemical-based biosensors. The information about the analytes is obtained by measuring the change in Faraday current or interfacial impedance by reduction or oxidation reaction (redox) in the specifically designed working electrode in the presence of a target analyte [61,62]. The optical detection of bioanalytes is one of the most sensitive and it can even detect single molecules. The optical mechanism includes surface plasmon resonance (SPR), which measures the local refractive index due to adsorptions of the targeted analytes [63,64]. The other optical mechanism is based on fluorescence resonance energy transfer (FRET), where nonradiative energy is transferred between two fluorophores via a pole–dipole coupling [65]. Zhu et al. found for the first time excellent fluorescence quenching ability of ssDNA in the presence of single-layer MoS_2_ nanosheets (NS) [66]. The van der Waals interaction between the dye-labeled ssDNA probe and MoS_2_ nanosheets almost entirely quenched the FL intensity of the probe once it adsorbed on the MoS_2_ surface. Upon hybridization of the ssDNA with complementary targeted DNA, hybridized dsDNA detached, resulting in restoring FL intensity by the FRET mechanism. The quantitative analysis of the restored FL intensity with the targeted DNA gives information about the concentration and the type of DNA. The specific binding nature of small-size ssDNA with the targeted DNA provides excellent selectivity and ultra-sensitivity.

By changing the probe ssDNA corresponding to different targets, sensing of different DNA as well as other biomarkers can be achieved [67,68,69]. While discussing electrical biosensors, we primarily focus on FETs based biosensors. FETs are present in every digital circuit, in gadgets ranging from computers, mobile phones, various sensors, and others. Thus, FET-based biosensors can be integrated into modern-day electronic circuits for the real-time detection of biomarkers. Recently, Park et al. demonstrated sensitivity enhancement by creating nanopores on the MoS_2_ surface to detect cortisol with a detection limit of 1 gM/mL in human serum and in an artificial saliva [70]. Details about the various types of biosensors based on TMDs will be discussed shortly. First, the review highlights various synthesis methods of 2D TMD nanostructures and then their application as biosensors based on (a) electrical, (b) electrochemical, and (c) optical mechanisms. Finally, a brief discussion on future prospects will be presented.

## 2. Synthesis of 2D TMD Materials

**Figure 2:**
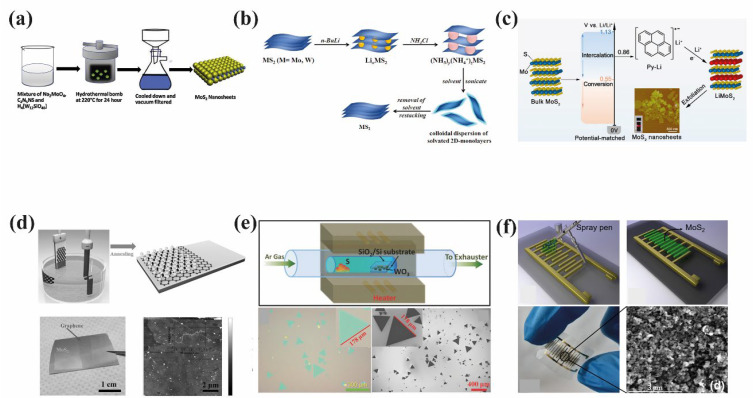
Synthesis/growth methods for 2D TMDs (**a**) Hydrothermal method, a bottom-up approach for synthesizing nanomaterials where the constituent atoms react to form thin films TMDs at high pressure and temperature. Reprinted with permission from ref. [87] copyright 2018 Elsevier; (**b**) Liquid exfoliation method is a top-down process. The schematic shows the ion intercalations-assisted liquid exfoliation of 2D NSs. The ions moving between the layers weaken the interlayer forces, and by agitating in solution, 2D nanosheets are obtained in suspension. Reprinted with permission from ref. [82]. Copyright 2014 American Chemical Society; (**c**) Electrochemical-assisted ion intercalations specific using redox potential. Reprinted with permission from ref. [83]. Copyright 2022 American Chemical Society; (**d**) Electrochemical deposition is cost-effective for the large-area synthesis of 2D TMDs. The schematic represents the heterostructure synthesis of graphene/MoS_2_ thin films. Reprinted with permission from ref. [88]. Copyright 2017 John Wiley and Sons; (**e**) Chemical vapor deposition (CVD) technique is a controlled synthesis method for high-quality 2D TMDs. The schematic shows the equipment setup along with optical images of 2D TMDs grown by CVD. Reprinted with permission from ref. [89]. Copyright 2013 John Wiley and Sons; (**f**) Printing of 2D TMD inks. TMD NSs can be dispersed in a liquid for various printing techniques, such as inject printing, and can be printed on a flexible substrate for wearable devices, reprinted from ref. [45]. Copyright 2022 under Creative Commons.

The growth/synthesis protocols of 2D TMDs play a significant role in their applications in electronic and optical devices, sensing, drug delivery, etc. A summary of growth methods is given in Figure 2. Top-down and bottom-up are two standard approaches for the synthesis of 2D nanomaterials. Nanomaterials are obtained by breaking down their bulk counterparts in top-down methods, whereas atomic range chemical or physical forces aid in the assembly of basic units in the formation of larger structures in bottom-up methods. Scotch-tape-aided mechanical exfoliation is a top-down method where thin layers of TMDs can be exfoliated from their bulk form. Graphene was first obtained from graphite flakes using scotch tape in 2004 [1]. The same method was later used for MoS_2_, WS_2_, MoSe_2_, and WSe_2_ as well [71,72,73,74,75,76,77,78,79]. This method is simple and cost-effective and helps in producing high-quality thin films; however, it suffers from the perspective of repeatability and uniformity over a large area. Thus, mass production for practical applications is not feasible. Other than mechanical exfoliation, liquid-based exfoliation includes chemical as well as physical techniques. For chemical exfoliation, interactions between the bulk material and some chemical agent produces 2D TMDs. Some examples include ion exchange [80,81,82], redox-based [83], surfactant-assisted methods, ion intercalations, electrophoresis, etc. [84]. Physical methods include tip sonication and ultrasonication-assisted exfoliation. Acquiring control over the lateral size and thickness is not straightforward with liquid-based exfoliating methods as in mechanical methods. In addition to these limitations, liquid-based methods leave impurities or by-products and lead to the creation of defects in the final 2D film. These defect sites are used for the bio-functionalization of the receptor for the selective capturing of analytes. Radiofrequency (RF) sputtering has also been used for the controlled deposition of large-area 2D TMDs. The films formed by RF magnetron sputtering consist of defects lacking crystallinity and quality, but thermal annealing was shown to improve the crystalline quality [85,86].

Bottom-up methods include hydrothermal synthesis, electrochemical deposition, chemical vapor deposition (CVD) and metal organic CVD or MOCVD. Hydrothermal synthesis of 2D materials is one of the easiest routes for 2D TMDs with low environmental impact and high-efficiency [87,90,91]. This method is very useful for synthesizing hybrid TMD composites with other nanomaterials for better performance [92,93]. The biggest challenge is control over the lateral size and achieving a uniform thickness of the TMD structures. However, the defect sites present in the final 2D TMDs obtained via hydrothermal processes prove useful for surface functionalization and sensing applications [94]. Electrochemical deposition is a highly controlled method for large-area synthesis of TMDs, such as MOS_2_ and WS_2_ [95,96]. The large areas of up to a few cm in size of MoS_2_ films were synthesized by electrochemical deposition by Wun et al. [88]. Atomic layer deposition (ALD) has also been used for the thin film deposition of TMDs [97,98,99]. ALD is well known for the uniform deposition of dielectric thin films [100,101]. By varying the cycle number, precursor gases, and substrate temperature, high-quality large-area TMD films have been obtained, which exhibited excellent opto-electronic properties [97,98]. Moreover, ALD is good for the formation of various heterostructures [98]. CVD yields large-area 2D TMD films with controlled lateral size and thickness. CVD-grown films are of the highest quality in terms of crystallinity and uniformity [36,89,102,103,104,105,106]. The basic principle of CVD is that thermal energy is used to induce chemical reactions between precursors in their vapor phases. A carrier gas is used for the deposition of 2D thin films onto suitable substrates. Large-area 2D heterostructures have been synthesized with superior optoelectronic properties using CVD [107,108]. Chubarov et al. have successfully grown monolayer WS_2_ over 2-inch sapphire substrates with a great uniformity [52]. The monolayer TMDs grown by CVD have shown excellent optoelectronic properties. Das et al. studied 2D TMD FETs extensively for future-generation applications in electronic as well as biomedical devices [25,26,109]. MOCVD is another technique for the synthesis of wafer-scale TMD films [110,111], which uses metal organic precursors. Inkjet printing of 2D TMDs has also been reported [54,112]. The advantages of printing include eliminating the need for conventional high-cost lithography and an overall low-cost process. However, TMD flakes are dissolved in a suitable solvent for ink preparation, which might leave solvent residue in the final device, limiting its overall performance. There are still challenges to the low-cost synthesis of 2D materials with control over their size and thickness. Extensive research is ongoing for the controlled synthesis of TMDs.

## 3. Biosensing Using 2D TMD Materials

### 3.1. Electrical Biosensors

**Figure 3:**
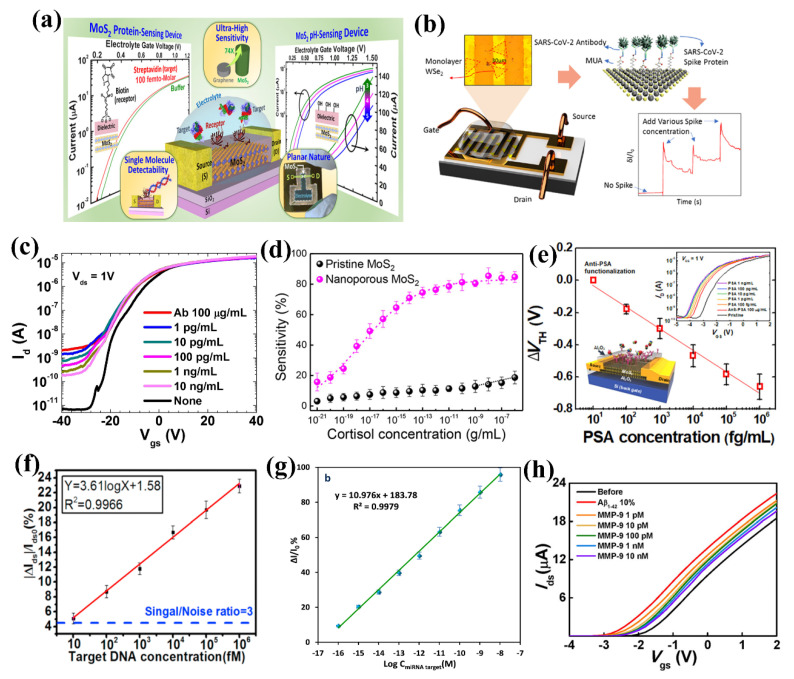
Electrical field-effect transistor-based biosensors (**a**) schematic of MoS_2_ FET for pH and streptavidin sensing. Schematic reprinted with permission from ref. [59]. Copyrights 2014 American Chemical Society; (**b**) Schematic of WSe_2_ FET for SARS-CoV sensing, reprinted with permission from ref. [115]. Copyrights 2021 American Chemical Society; (**c**) Transfer characteristics of MoS_2_ FET at different concentrations of prostate-specific antigen (PSA), reprinted with permission from ref. [57]. Copyright 2014 under Creative Commons; (**d**) Sensing response of MoS_2_ FET with and without nanopores for cortisol detections, reprinted with permission from ref. [70]. Copyrights 2022 American Chemical Society; (**e**) Multi-layer MoS_2_ FET for PSA sensing, reprinted with permission from ref. [116]. Copyrights 2017 American Chemical Society; (**f**) DNA detection response of MoS_2_ FET biosensor, reprinted with permission from ref. [29]. Copyright 2018 Elsevier; (**g**) Calibration curve of miRNA-155 detection using MoS_2_ FET, reprinted with permission from ref. [56]. Copyright 2022 Elsevier; (**h**) Transfer characteristics of MoS_2_ for the detection of circular protein, reprinted with permission from ref. [117]. Copyrights 2019 American Chemical Society.

Electrical sensors transform the information about the analytes into useful user-readable electrical signals, such as current or resistance change. This section will discuss the historical progress of TMD-based FET biosensors and the current challenges. The basic working principle of FETs is that the current between the source and drain terminals is controlled by a third terminal called the gate. FETs are essential components of any modern-day electronic circuit and have well-established technologies for fabrication. Thus, electrical detection has the potential for integration with various modern-day electronic devices for real-time detection of bioanalytes. The electrical biosensors consist of a semiconductor transducer element along with a bioreceptor connected to the transducer. The bio-analytes interact with the semiconducting transducer element through the receptor and change the transducer’s electrical properties by the charge transfer mechanism. The attachment of bio-analyte to the semiconductor surface is equivalent to a potential bias at the gate terminal and hence, leads to a change in the drain current. The main advantage of FET biosensors is the enhancement of sensitivity and limit of detection (LoD). Table 1 provides a summary of FET-based TMD biosensors, and Figure 3 captures a cross-section of the FET sensors discussed in this review.

#### 3.1.1. Biomarker Detection

Biomarkers are biological markers for a specific medical state that can be observed or measured externally. They are direct outcomes of specific biological, pathogenic, or pharmacological processes [113]. Detection of biomarkers is crucial in disease diagnosis, state of progress, and treatments. Detection at ultralow concentration identifies the disease onset early, leading to a higher chance of treatment and recovery. Two-D TMDs, with their high density of active sites, give ultrasensitive responses and ultralow LoD [63]. For example, cancer is one of the deadliest diseases, and a large number of cancer patients can be treated successfully if detected at an early stage of the disease. According to estimates by the International Agency for Research on Cancer, there will be 18.1 million new cancer cases and 9.6 million cancer-related deaths. Lung cancer is the deadliest, followed by breast and prostate cancer [114].

Prostate cancer is one of the most common cancer diseases for males, and prostate-specific antigen (PSA) is a well-studied biomarker for prostate cancer [118]. Detection of PSA at low concentrations is highly desirable, and Wang et al. demonstrated biomodifications of multilayer MoS_2_-based microfluidic FETs for specific detection of PSA. The MoS_2_ flakes were mechanically exfoliated on a SiO_2_ substrate and passivated by HfO_2_ before functionalizing with anti-PSA to have specific interactions. The concentration-dependent analysis shows a sub-pM sensitivity with a detection limit of 375 fM [79]. The specific binding of PSA on the functionalized surface shows no response with bovine serum albumin (BSA), implying the highly selective detection of PSA. An ultra-thin layer of HfO_2_ helps biofunctionalization and protects the MoS_2_ layer from direct interaction with the aqueous medium. This study opened a new pathway for MoS_2_-like materials application in FET-based biosensing. Lee et al. functionalized the MoS_2_ surface directly by anti-PSA, utilizing the hydrophobic nature of MoS_2_. The attachment of positively charged anti-PSA antibodies is equivalent to applying a positive bias and hence, modulates the off-state current more significantly. A significant number of electrons was injected into the MoS_2_ channel once anti-PSA adsorbed on the surface. The direct functionalization of the MoS_2_ surface enhanced the lower detection limit to 1 pg/mL and reduced the fabrication complexity [57]. Yoo et al. fabricated MoS_2_ FET on a flexible polyimide substrate integrated with a LED-based readout system for the detection of PSA. This study showed that the MoS_2_ FET is highly stable under mechanical stress even after 10,000 bending cycles [119]. Park et al. studied the detection of PSA in a dry medium. Anti-PSA was attached to the surface with the help of AuNPs, which enhanced the sensitivity many times. The non-specific response was minimized by integrating blocking agent casein, which improved the selective response, recording a detection limit of 100 fg/mL [116]. Hossain et al. fabricated WSe_2_-based FET for ultra-low detections of PSA. The biosensor has a linear response with a wide range of concentrations from 10 fg to 1 ng per ml with an exceptional sensitivity [60]. Sensitivity tests in complex biofluids, such as human serum samples, were not verified in the above-mentioned studies. Sensitivity toward biosamples is crucial for real-time PoC devices.

MicroRNAs are common breast cancer biomarkers used for diagnosis [120]. Majd et al. used chemically synthesized MoS_2_ in a FET to detect miRNA-155 in human serum and breast cancer cell line samples toward PoC devices. The surface was functionalized with amino-modified probe RNAs through physical adsorption, which reduced the channel conductivity. In the presence of targeted RNA, the probe hybridized and detached from the surface, increasing the channel conductivity. A linear response over 0.1 fM to 10 nM was achieved with a detection limit of 0.03 fM at the optimized conditions. The linear response with concentrations of miRNA-21 in human serum is a significant step toward PoC systems [56]. Circulating protein (CP) is essential for diagnosing and treating cancer patients. Park et al. employed scotch-tape-assisted transfer of MoS_2_ onto an Al_2_O_3_-deposited substrate for the FET-based matrix metalloproteinase-9 (MMP-9) sensor and achieved a detection limit of 1 pM [117]. The same group has also used multilayer MoS_2_ functionalized with aptamer to detect cortisol in human serum and artificial saliva [70]. Sulfur vacancies aid in the functionalization of the bioreceptor and hence, the sensitivity. Additional sulfur defects by creating the nanopore on the MoS_2_ surface enhanced the sensitivity exceptionally. The nanopore-modified MoS_2_ has the lowest detection limit of 1 ag/mL with a wide range of sensitivity from ag/mL to µg/mL.

#### 3.1.2. Detection of Bacteria and Virus

Bacteria and viruses are microorganisms living everywhere, including air, contaminated food, water, body fluids, etc. Some are beneficial to human health, while others are detrimental [121]. Bacterial and viral infections are mostly transferred via physical contact and non-contact spreading. The rapid spreading of these infections causes millions of deaths worldwide [122]. Thus, rapid detection of bacterial and viral infections is vital in human health and environmental monitoring. Detection of bacteria and viruses using TMDs is still in the developing stage. Graphene and reduced graphene oxide (rGO) have been studied in the FET-based detection of bacteria and viruses [123,124,125,126,127]. TMD-based FET biosensors for detecting bacteria and viruses are limited. Moudgil et al. have shown highly selective and sensitive detection of Gram-positive bacteria based on MoS_2_/TiO_2_ hybrid nanostructure FET [128]. The TiO_2_ surface functionalized with vancomycin was able to differentiate between Gram-positive and negative bacteria. A sensitivity of 49% was observed toward S. Aurus with a dynamic response range between 50–10^6^ cfu/mL with a detection limit of 50 cfu/mL. The first WSe_2_-based FET biosensor for virus detection was reported by Fathi-Hafshejani et al. [115], wherein monolayer WSe_2_ was functionalized by SARS-CoV-2 antibody 11-mercaptoundecanoic acid (MUA) for the real-time detection of SARS-CoV-2. Selenium vacancies present in WSe_2_ help in covalently bonding with MUA. The ability of functionalization of monolayer WSe_2_ also opened avenues for the usage of other TMDs for different pathogenic bacteria and viruses. The MUA functionalized WSe_2_ FET was able to detect as low as 25fg/µL in real-time.

#### 3.1.3. Detection of DNAs

Detection of DNA is crucial for disease diagnosis, drug delivery, food quality monitoring, environment monitoring, etc. The polymerase chain reaction (PCR) is the most trusted and standard DNA amplification and identification method, but its high cost and time remain a drawback. There is a need for a fast and cost-effective way of DNA sequencing and identification. Lee et al. used chemically synthesized MoS_2_-based FET sensors for the selective detection of DNA molecules. The van der Walls interaction allows direct functionalization of the MoS_2_ surface by probe ssDNA. Adsorption of negatively charged ssDNA on the basal plane of MoS_2_ reduces the effective positive gate voltage, reducing drain current significantly. On adding the targeted DNA, the probe DNA hybridized to dsDNA and detached from the MoS_2_ surface, increasing its conductivity. At optimum conditions, the sensing device has a highly selective response over 10 fM to 10 nM of complementary DNA [129]. The absence of dangling bonds ensures stability over different pH environments. Mei et al. reported MoS_2_ based FET sensor for the detection of targeted DNA by using phosphorodiamidate morpholino oligos (PMO) as a probe. The strong interaction between the PMO and targeted DNA improves the detection limit to 6 fM, compared to previous reports. Owing to its low detection limit and ability to detect different concentrations of targeted DNA in human serum, it is a step forward for the PoC diagnostics [29]. Liu et al. used monolayer MoS_2_ grown by CVD in bio-FETs for the detection of specific DNA targets. Thiol-modified ssDNA probes functionalize the MoS_2_ surface by using AuNPs through strong Au–SH bonding that enhances the sensitivity by many folds. The monolayer MoS_2_ can selectively detect 100 aM complementary targeted DNA [36]. Bahri. et al. used WS_2_ for the first time for the detection of DNA hybridization. The CVD monolayer WS_2_ was functionalized with an ssDNA probe by van der Walls interaction. The unreacted WS_2_ surface was blocked by blocking agent poly-C (C-15) to eliminate the non-specific binding for better selectivity. This DNA sensor has an excellent linear response over 0.1 fM to 1 nM DNA concentration with a detection limit of 3 aM [130].

Sarkar et al. fabricated pH and streptavidin sensors based on functionalized MoS_2_ FET. Lowering the solution pH increases positive ion concentration, which is equivalent to applying positive potential at the gate terminal. The biosensor has a linear response over 3 to 9 pH values and hence serves as a reliable pH sensor. For specific detection of streptavidin, the MoS_2_ surface was functionalized with biotin. The functionalized MoS_2_ sensor is highly selective to streptavidin with a detection limit of 100 fM [59]. The semiconducting MoS_2_ has enhanced the sensitivity by almost 74-fold over graphene. Man et al. fabricated a few layers of MoS_2_ FET by SF_6_-assisted plasma etching from bulk MoS_2_ and passivated it by HfO_2_. They functionalized the HfO_2_ by anti-human tumor necrosis factor–alpha (TNF-α) antibody corresponding to the TNF-α biomarker. At the optimum sensing environment, the device showed a linear response over 60 fM to 6 pM concentrations and a detection limit of 60 fM in linear and subthreshold regimes. The statistics over several devices showed an excellent repeatability [131]. Nam et al. compared electrical sensing of TNF-α and Streptavidin using MoS_2_ and WSe_2_ FET biosensors [132], and both TMDs exhibited similar detection limits. Chen et al. detected kanamycin (KAN) using an aptamer functionalized MoS_2_ FET biosensor. The highly specific interaction between the probe aptamer and KAN shows a selective response toward KAN. Surface modification by AuNPs enhances probe attachment and sensitivity [133].

**Table 1:** Summary of TMD based electrical biosensors.

Matrix	Method	Target Analyte	Linear Range	LoD	Reference
Multi-layer MoS_2_	FET based	PSA		375 fM	[79]
Multi-layer MoS_2_	FET based	PSA	1 pg/mL–10 ng/mL	1 pg/mL	[57]
Multi-layer MoS_2_	FET based	PSA	1 pg/mL–1 ng/mL	1 pg/mL	[119]
Multi-layer MoS_2_	FET based	PSA	100 fg/mL–1 ng/mL	100 fg/mL	[116]
Multi-layer WSe_2_	FET based	PSA	10 fg/mL–1 ng/mL	10 fg/mL	[60]
Multi-layer MoS_2_	FET based	miRNA-155	0.1 fM–10 nM	0.03 fM	[56]
Multi-layer MoS_2_	FET based	Circulating protein	1 pM–10 nM	1 pM	[117]
Multi-layer MoS_2_	FET based	cortisol	1 ag/mL–1 µm/mL	1 ag/mL	[70]
MoS_2_/TiO_2_	FET based	S. Aurus	50–10^6^ cfu/mL	50 cfu/mL	[128]
Monolayer WSe_2_	FET based	SARS-CoV-2	25 fg/µL–10 ng/µL	25 fg/µL	[115]
Multi-layer MoS_2_	FET based	DNA	10 fM–10 nM	10 fM	[129]
Few-layer MoS_2_	FET based	DNA	10 fM–1 nM	6 fM	[29]
Monolayer MoS_2_	FET based	DNA	100 aM–100 fM	100 aM	[36]
Monolayer WS_2_	FET based	DNA	0.1 fM–1 nM	3 aM	[130]
Multi-layer MoS_2_	FET based	Streptavidin		100 fM	[59]
Multi-layer MoS_2_	FET based	TNF-α	60 fM–6 pM	60 fM	[131]
Multi-layer MoS_2_ and WSe_2_	FET based	TNF-α and Streptavidin	60 fM–6 pM and 70 fM–70 pM	60 fM and 70 fM	[132]
Monolayer-bilayer	FET based	kanamycin	1 nM–100 µM	0.66 nM	[133]
MoS_2_/rGO	FET based	H_2_O_2_	1 pM–100 nM	1 pM	[134]

Reactive oxygen species, such as H_2_O_2_, play a vital role in the functioning of cells and neuro systems. Detection of such species is essential for the continuous monitoring of cell functioning. Zheng et al. demonstrated the real-time detection capability of H_2_O_2_ in HeLa cells using MoS_2_/RGO heterostructure FET. The sensitivity toward H_2_O_2_ increased significantly compared to pristine RGO. Hela cells generate H_2_O_2_ when reacted by phorbol 12-myristate 13-acetate (PMA) and detected by the FETs, proving the capability to detect H_2_O_2_ in complex biofluids [134].

TMD-based FET biosensors are still new in the research domain compared to well-established silicon technologies. Generally, TMDs grown at high temperatures lead to degradation of the growth substrate. One major disadvantage of this for electrical biosensors is that the 2D TMD films need to be transferred from the growth substrate to the device substrate. The transfer process is complex and chemical residue is present on the transfer samples, leading to lower quality and performance issues. There is a need for a low-temperature growth mechanism or improved transfer methods to mitigate this problem in electrical biosensors.

The charge screening effect in FET devices, defined by the Debye length, remains a big challenge. This is the maximum distance from the sensing channel, where analytes can modulate the channel conductivity. As the ionic strength increases, the Debye length decreases. The complex and high ionic strength biofluids limit the Debye length to a few nm. Although TMDs have been successfully demonstrated as selective biosensors, most studies have been done in controlled environments, and further development is required in terms of rigorous testing in complex media. Thus, applying TMD-based FETs as state-of-the-art PoC devices has a long way to go.

### 3.2. Electrochemical Biosensors

Electrochemical sensors are three electrode-based sensing platforms where the electrochemical reaction reduction–oxidation (redox) creates ions and charges, changing the electrochemical response. The three electrodes are the working electrode (WE), the reference electrode (RE), and the counter electrode (CE). All the potentials are measured with respect to RE, while CE completed the electrochemical cell connections. The WE is modified by nanomaterials for better electrochemical activity. The basic operating principle is the detection of Faraday current using voltammetry or amperometry and the modulation of interfacial impedance by electrochemical impedance spectroscopy (EIS). The WE is modified with suitable biorecognition elements for detecting specific analytes via redox reactions, which generate or suppress electrons or ions and change the current across the WE. In the case of EIS, the analytes adsorbed on the modified WE surface modulate the electrochemical current or interfacial impedance. The quantitative correlations between the change in the interfacial impedance or electrochemical current with the number of analytes give the sensing response.

#### 3.2.1. Detection of Biomarkers

Dopamine (DA) is a neuro biomarker and plays an important role in the functioning of the neurological system. Sakthivel et al. decorated cobalt oxide polyhedrons on an MWCNT/MoS_2_ hybrid system by conventional hydrothermal method for electrochemical detection of DA [135]. The modified electrode showed great stability, high sensitivity and selectivity, and a low detection limit of 13 nM. The electrode retained 97% of its initial current even after 30 weeks of use, indicating its robustness. The feasibility of real-time application was tested with physiological samples, such as rat and human serum, in an optimized lab environment. As discussed earlier, miRNAs are cancer biomarkers. Su et al. used gold nanoparticle (Au NPs)-modified MoS_2_ to detect miRNA-21 by EIS and differential pulse voltammetry (DPV) [136]. The results showed selective detection of miRNA-21 in the fM range for both methods. The miRNA-21 was added to human serum for accurate sample detection and studied with the same device. The results indicated that the Au NP decorated MoS_2_ was an excellent real-time detector for miRNA-21. Zhu et al. demonstrated thionine-reduced AuNP functionalized MoS_2_ sheets for electrochemical detection of miRNA-21 with a detection range of 1 pM to 10 nM and a detection limit of 0.26 pM [61]. Chand et al. synthesized copper ferrite-decorated MoS_2_ nanosheets functionalized with thiol-modified biotin for microfluidic-based electrochemical sensors to detect paratuberculosis-specific miRNAs [137]. Paratuberculosis (pTb), or Johne’s disease, is a deadly disease in dairy cattle as it is highly contagious and asymptomatic [138]. The microfluidic assisted sensing allows the screening of multiple samples simultaneously. The optimized conditions showed real-time detection of miRNA at the lowest concentration of 0.48 pM with excellent selectivity. The complex biological fluid analysis of infected blood and fecal samples helps in the detection of miRNA. This sensor also successfully detected actual samples from infected cows. Carcinoembryonic antigen (CEA) is a cancer biomarker produced by colorectal cancer and found at very low concentrations [139]. Thus, sensitive and low-level detection of CEA is vital for the early detection and treatment of colorectal cancer. Wang et. al. synthesized a nanocomposite of flower-like MoS_2_ with rGO and Ag NPs for ultrasensitive detection of CEA [140]. Incorporating Ag NPs in MoS_2_-GO composite enhances the electrochemical activity manyfold because the synergistic effect between MoS_2_ and Ag NPs improves the sensitivity. The wide range of detection is from 0.01 pg/mL to 100 ng/mL with a detection limit of 1.6 fg/mL, which is much lower than the clinically safe limit.

Liu et al. reported an AuNP-decorated MoS_2_/T_3_C_2_ hybrid structure for ultrasensitive electrochemical sensing of miRNA-182, a well-known lung cancer biomarker [141]. The thiol-modified probe ssRNA was mobilized by the hybrid structure of the well-known Au–SH solid bond. Because of its negative charge, the electrochemical activity of the hybrid decreased after the ssRNA modification. The binding of the targeted miRNA-182 with the probe RNA resulted in dsRNA hybrids, which, when released from the Au NPs, increased the electrochemical activity. At optimum conditions, quantitative analysis of the RNA hybridization gave a linear detection range of 1 fM to 0.1 nM with a detection limit of 0.43 fM. MicroRNA-155 is a cancer biomarker found in ultra-low concentrations in body fluids, such as plasma, at the early stage of cancer patients [142]. Liu et al. studied the electrochemical sensing of miRNA-155 using MoS_2_ thin films deposited by ALD and modified by AuNPs [143]. For the selective detection of miRNA-155, thiol-modified probe RNA was attached to the MoS_2_ surface via SH-Au chemistry. To avoid a non-specific response, unbounded Au NPs were blocked by blocking agent 6-mercaptohexanol (MCH). Cyclic voltammetry (CV) and EIS measurements of the modified electrode at different stages of the fabrication show that the Au NPs enhanced the electrical conductivity by synergistic effect and enhanced electron transfer process. After functionalization and blocking of the unreacted sites of Au NPs, the electrical conductivity decreased as the charge transfer was hindered. For the concentration-dependent study, toluidine blue (TB), a phenothiazine dye, was used as the hybridization monitor owing to the presence of π-π conjugate electron. The sensors could detect miRNA-155 ranging from 1 fM to 10 nM with a detection limit of 0.32 fM. This study indicates that the ALD-deposited MoS_2_ has the potential to detect actual biological samples in real time. Rawat et al. fabricated MoS_2_ based electrochemical biosensor to detect and quantify glutathione (GSH), a cancer biomarker [144]. For the binding of GSH, the glutathione-S-transferase (GST) enzyme was used for the catalytic reduction of GSH. The sensing platform showed excellent sensitivity of 700 pA/µM with a linear response from 10 µM to 500 mM concentration of GSH.

#### 3.2.2. Detection of Bacteria and Virus

Hepatitis B virus (HBV) infection is one of the serious public health concerns and can cause some deadly diseases, such as cirrhosis and hepatocellular carcinoma (HCC) [145]. Hepatitis B e antigen (HBeAg) is one of the most reliable tumor biomarkers for identifying HBV infections. Gao et. al. developed an electrochemical immunosensor based on gold@palladium (Au@Pd) NP decorated MoS_2_ functionalized multiwall carbon nanotubes (Au@Pd/MoS_2_@MWCNTs) [62]. The MoS_2_/MWCNTs hybrid composite enhanced electrochemical activity, and the incorporated Au@Pd NPs amplified the sensitivity towards HBeAg detection by synergistic effect. The sensor showed a systematic current increase with the addition of HBeAg ranging from 0.1 pg/mL to 500 pg/mL and had a detection limit of 26 fg/mL.

#### 3.2.3. Detection of DNAs and Other Bio-Analytes

**Figure 4:**
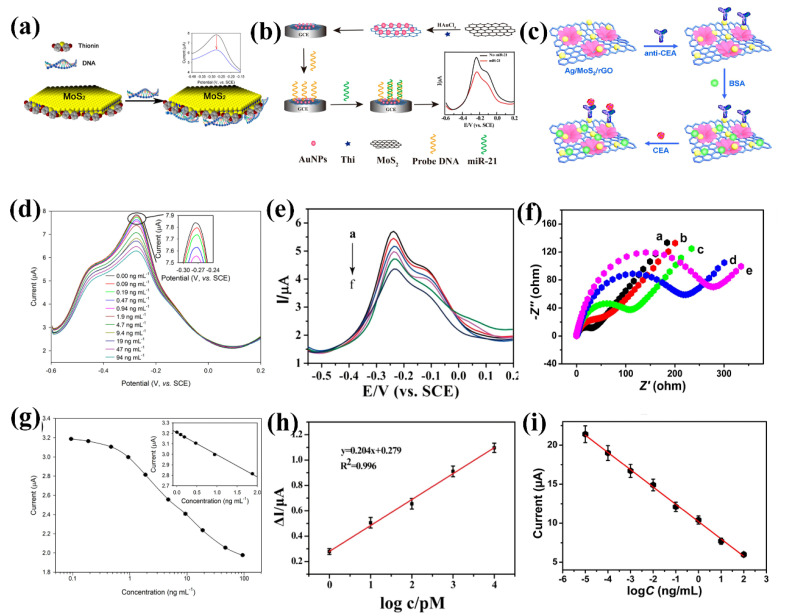
(**a**) Electrochemical DNA sensing using MoS_2_, schematics representations for functionalization of MoS_2_; (**b**) Label-free electrochemical detection of miRNA-21 using Au NP decorated MoS_2_ NSs, schematic representations for working electrode modifications; (**c**) Ag NPs decorated MoS_2_/rGO hybrid NSs for electrochemical sensing of carcinoembryonic antigen schematic representation; (**d**) electrochemical sensing response at different concentrations of DNA; (**e**) electrochemical response at different concentrations of miRNA-21 in the electrochemical cell; (**f**) Nyquist plots; (**g**) calibration curve for DNA detection; (**h**) miRNA-21 calibration curve; (**i**) calibration curve for CEA detection. (**a**,**d**,**g**) reprinted with permission from ref. [146], Copyrights 2014 American Chemical Society. (**b**,**e**,**h**) reprinted with permission from ref. [61], Copyrights 2017 American Chemical Society. (**c**,**f**,**i**) reprinted with permission from ref. [140], Copyright 2018 Elsevier.

Figure 4 captures representative DNA detection using an electrochemical approach. Wang et al. demonstrated electrochemical sensing of dsDNA using thionin-functionalized MoS_2_ sheets as a working electrode. The electrostatic attraction of thionin to the MoS_2_ defect sites was confirmed by X-ray photoelectron spectroscopy (XPS) analysis and the redox peak at −0.27 V at square wave voltammetric measurements [146]. The redox peak current at −0.27 V decreased consistently with increasing concentration of dsDNA in the solution. The linear response was obtained from 0.09 to 1.9 ng per ml dsDNA with a detection limit of 0.09 pg/mL. Redox current decreased after the addition of complex biofluid, circulating DNA extracted from human serum, demonstrating its capability as a PoC device. Yang et al. synthesized ZnO/MoS_2_ hybrid nanocomposite for the electrochemical sensing of DNA with great sensitivity [147]. The hybrid composite not only helps in direct charge transfer but also in probe immobilization. The probe ssDNA is attached to the nanocomposite surface by electrostatic interaction between positively charged ZnO and negatively charged ssDNA. At an optimum environment, the DPV measurement of the modified electrode with different concentrations of promyelocytic leukemia (PML) and retinoic acid receptor alpha (RARA) exhibits a linear response over a wide range. The low detection limit of 0.66 fM demonstrates its potential for ultralow detection of DNA by the heterostructure.

Zhang et al. fabricated poly-xanthurenic acid (PXA) functionalized MoS_2_ electrochemical biosensors to detect circulating tumor DNA in blood samples [148]. The novel polymer XA has low toxicity, acceptable redox activity and good electrochemical performance. The probe ssDNA was immobilized on hybrid PXA/MoS_2_ nanostructure by π-π conjugate interactions. The immobilization of ssDNA on the surface decreased the current drastically due to charge transfer and blocking. With the addition of the targeted circulating tumor DNA, the ssDNA hybridized to form dsDNA. The weak interaction between PXA and dsDNA releases the DNA from the surface, restoring electrochemical activity. The quantitative analysis of the current restoration with DNA concentration shows a linear response range of 0.1 fM/l to 100 pM/l with a detection limit of 0.018 fM/L. The sensor shows excellent reproducibility. The van der Waals interaction between the ssDNA with MoS_2_ nanosheets was utilized by Zhou et al. for electrochemical sensing of Kanamycin, the widely used antibiotic for bacterial infections and tuberculosis [149]. In the presence of biotin-modified assist DNA, probe DNA and aptamer DNA formed a Y shape dsDNA structure, which has a very low adsorption probability with MoS_2_ sheets. The addition of Kanamycin aided in the binding with the aptamer DNA and breaking the Y shape of dsDNA to ssDNAs. The MoS_2_-modified glassy carbon electrode (GCE) adsorbed the ssDNA. The interactions between the biotin and streptavidin enhanced the mobilization of the biotin-modified assist DNA and probe DNA hybrid on MoS_2_-modified GCE. The catalytic effect between alkaline phosphatase and p-nitrophenol phosphate produced p-nitrophenol (PNP), an electrochemically active molecule. The quantitative analysis between the Kanamycin concentration and peak oxidation current showed a linear response from 0.1 nM to 100 nM. The threshold limit of detection was 0.03 nM. The Kanamycin in Kanamycin Sulfate Eye Drops was successfully detected with the sensor with less than 9% variation and remarkable selectivity. Zhang et al. synthesized WS_2_/graphite microfiber hybrid for the electrochemical detection of adenine and guanine. WS_2_ synthesis directly on graphite microfiber exhibited excellent charge transfer characteristics and electrocatalytic oxidation response. The adsorption of adenine and guanine on the WS_2_ surface changed its electronic and charge transfer properties. The CV analysis showed two oxidation peaks at +0.73 V and +1.03 V, corresponding to the oxidation of adenine and guanine, respectively. The concentration-dependent CV response showed a systematic increase in the reduction current due to charge transfer resulting from the adsorption on the WS_2_ surface. The electrochemical sensor had a linear response ranging from 0.5 µM to 20 µM concentration of adenine and guanine [150].

The partial reduction of oxygen produces H_2_O_2_ during various biological processes, which plays an essential role in signal processing and transduction of cells [151]. The imbalanced ions affect the stress in cells and eventually lead to several diseases, including cancer [152]. Wang et al. used MoS_2_ nanoparticles to detect H_2_O_2_ at the nM level without using any enzymes [153]. The high density of electroactive sites of MoS_2_ NP increases the electrochemical reduction of H_2_O_2_ and hence, improves the sensitivity along with a low detection limit. Ma et al. demonstrated H_2_O_2_ and cholesterol sensing by oxidized glutathione-modified MoS_2_ (MoS_2_-GSSG) NSs. The high affinity of MoS_2_-GSSG towards the 3,3′,5,5′-tetramethylbenzidine (TMB) substrate ensures uniform distribution. Peroxidase-like catalytic activities of MoS_2_-GSSG NSs convert the H_2_O_2_ into ^*^OH, which oxidizes the TMB [154]. With this catalysis-based reaction, the detection limit was as low as 0.5 µM. Shu et al. synthesized nanoflower-like interlayer expanded MoS_2_ (IE-MoS_2_) and nonexpanded MoS_2_ (NE-MoS_2_) by thiourea-assisted hydrothermal route for electrochemical sensing of the H_2_O_2_ [155]. The GCE was modified by IE-MoS_2_ and NE-MoS_2_ to check the electrochemical performance for H_2_O_2_ detection. The IE-MoS_2_ modified electrode showed an enhancement in current with good sensitivity as it promoted the reduction of H_2_O_2_ to OH^−^. Linear response with H_2_O_2_ concentration ranging from 2.3 × 10^−1^ to 14.2 × 10^3^ μM was reported with remarkable selectivity in the presence of ascorbic acid (AA), glucose, sucrose, uric acid (UA), dopamine (DA), NaCl and KCl. Real-time detection of H_2_O_2_ was carried out in complex biological fluids, including living cancer cells, e.g., human breast cancer cells (MCF-7), for PoC applications.

Diabetes is one of the most common medical issues worldwide, causing many serious health problems. Blood glucose is the critical parameter for monitoring diabetes. Hence, continuous blood glucose monitoring is crucial [156]. Currently, the commercially available PoC system can detect glucose in human blood drawn by an end user from 1 to 27 mM [157]. Instead of using a blood sample, the glucose level can also be detected by the patient’s sweat and saliva, which is a non-invasive technique. However, the glucose level in sweat and saliva is in the order of µM. Thus, we need ultrasensitive sensing devices to detect glucose in sweat and saliva, which is much lower than the sensing capabilities of enzyme-based commercial systems [156,157,158,159]. TMDs have the potential for lower detection limits and can detect glucose even in the nM concentration [160]. Su et al. decorated MoS_2_ NSs with Au@Pt core-shell nanoparticles for the electrochemical detection of glucose in the human serum [161]. The synergistic effect of the nanoparticles enhanced the electrocatalytic activity of the modified electrode towards glucose reduction, improving the sensitivity. Glucose detection in human serum with excellent recovery and accuracy implies its application in real-time in biofluids. The low detection limit of 1.08 µM shows the capabilities for ultralow glucose detection.

From the detailed account above, we can see that electrochemical biosensors with working electrodes modified by 2D TMDs have certainly improved the detection limits with enhanced specificity. Table 2 provides a summary of TMD-based electrochemical sensors. Commercialization of PoC electrochemical devices based on TMDs has not been possible to date. The current PoC devices for blood glucose monitors and reactive ion species detectors are fully enzyme based, lagging in terms of detection limits besides being quite expensive. The utilization of high active site densities of TMDs has enhanced the detection limit, but the working environments, such as specified pH and temperature-dependent response, have restricted commercialization. Desorption of by-products is essential for reusability, which may reduce the effective cost. Synthesis methods and functionalization can tune the properties of the TMDs specific for the analyte of interest towards PoC detection.

### 3.3. Optical Biosensors

Optical biosensors utilize the advanced and superior optical properties of 2D TMDs to detect bioanalytes. They are some of the most sensitive biosensors and can detect even a single bioanalyte in real-time. The typical mechanisms for detection in optical biosensors include surface-enhanced Raman spectroscopy (SERS), surface plasmon resonance (SPR), fluorescence imaging, and Forster resonance energy transfer (FRET). SERS measures the enhancement of the Raman signal of the samples after and before analyte adsorption on a SERS substrate. SPR strongly depends on the refractive index. Upon adsorption of the bio-analyte, there is a local change in the material’s refractive index. FRET is a nonradiative energy transfer mechanism between two fluorophores through dipole–dipole coupling. Due to its inverse sixth power law dependence, it is extremely sensitive to changes in the distance between fluorophores. Hence, modulation in the fluorescence intensity in the presence of a target analyte has been extensively used for the purpose of detection.

#### 3.3.1. Biomarker Detection

Biomarkers, as mentioned earlier, are direct outcomes of specific biological, pathogenic, or pharmacological processes [113]. 2D TMDs, with their high density of active sites, give ultrasensitive responses as biosensors used for biomarker detection [63]. Kong et al. carried out real-time detection of a prostate cancer biomarker in human serum for the first time using aptamer-functionalized MoS_2_ nanosheets. When added to the dye-labeled ssDNA aptamer, there is high fluorescence (FL) quenching of MoS_2_ NSs. This phenomenon was utilized for the purpose of sensing [162]. The adsorption of the target-specific ssDNA on the MoS_2_ surface almost entirely quenches its FL spectra due to charge transfer by van der Waals interaction. The addition of the target DNA leads to the binding with probe aptamers, and thus, desorption from the MoS_2_ surface restores the FL intensity. The quantitative study between the FL restoration and the targeted aptamer addition shows a linear response from 0.5 to 300 ng/mL with a detection limit of 0.2 ng/mL. With its high stability and specificity to aptamers, the sensor had a very high selectivity toward targeted DNA. The ability to detect PSA in complex biofluid human serum in real-time ensures its capability to be used as a PoC device. Dhenadhayalan et al. studied the molybdenum (Mo) series of 2D materials from MoO_3_, MoS_2_ to MoSe_2_ NSs for real-time detection of PSA by FL quenching [163]. Among those NSs, MoO_3_ had the lowest detection limit of 13 pM, whereas MoS_2_ and MoSe_2_ yielded 72 and 157 pM, respectively. The low detection limit of MoO_3_ NSs was attributed to the relatively electronegative element O as compared to S and Se. These ultrasensitive biosensors have the potential for practical use at preliminary testing facilities. Similar to prostate cancer in males, breast cancer is the most common cancer in women and shares a high percentage among all cancer-related deaths [114]. MicroRNAs are common biomarkers for breast cancer and are used for disease diagnosis [120]. Xi et al. first showed the detection of microRNA-21 (miRNA-21) using WS_2_ NSs through duplex-specific nuclease signal amplification (DSNSA) [164]. The binding of the dye-labeled ssDNA onto the basal plane of WS_2_ NSs quenches its FL intensity by almost 97%, indicating strong charge transfer between aptamers and WS_2_ NSs. Binding with the targeted miRNA-21, the probe DNA formed a DNA/RNA heteroduplex which acts as the substrate for duplex-specific nuclease (DSN) cleavage. The DSN selectively cleaves the ssDNA from the heteroduplex, allowing the miR-21 to hybridize with another ssDNA. This stimulated process improved the detection limit to 300 fM with extremely high selectivity and even allowed to differentiate a single mismatch RNA.

MiRNA extracted from various cancer cell detection shows excellent agreement with the quantitative real-time polymerase chain reaction (qRT-PCR) results, hence having the capability to use in PoC detection systems. Chi et al. demonstrated miRNA-21 sensing by ssDNA aptamer functionalized MoS_2_ NSs via an excellent FL quenching [165]. As reported previously, the FL of MoS_2_ NSs quenched on the adsorption of ssDNA on its surface. The sensing platform had a detection limit of 500 pM and distinguished even a single mismatch miRNA-21. The detection of miRNA-21 with human serum in different concentrations was demonstrated in a real-time analysis as a PoC system. Gómez et al. showed a red shift in the photoluminescence (PL) spectra of monolayer MoS_2_ when used for miRNA-21 detection. The thiol-modified probe ssDNA binds to the S vacancy sites and enhances the PL intensity because of charge transfer between them [166]. When the probe DNA captures complementary targeted DNA, it forms dsDNA, the PL peak shifts towards the lower energy region, and PL is quenched. Further study is needed for the concentration dependence of PL peak shift to find the sensitivity and detection limit. Lung cancer, as mentioned earlier, does not show symptoms and the biomarkers are at ultralow concentration. To detect, we need biosensors with ultralow lung cancer biomarker detection capability [114]. Cytokeratin 19 fragment (CYFRA21-1) is a well-studied biomarker for lung cancer [167]. Chiu and Yang used the principle of SPR with carboxyl-functionalized MoS_2_ (carboxyl-MoS_2_) to detect CYFRA21-1 in the human serum [63]. The sulfur vacancies in MoS_2_ act as attachment sites for the carboxyl group, making them more sensitive to incident light. The study showed that carboxyl-functionalized MoS_2_ SPR biosensor could detect breast cancer biomarker CYFRA21-1 from 0.05 pg/mL to 100 ng/mL with a detection limit of 0.05 pg/mL. The concentration-dependent measurement of CYFRA21-1 in human serum showed a detection limit corresponding to 3.125% CYFRA21-1 in human serum. Zhao et al. detected the tumor biomarker CEA by FL quenching of MoS_2_ NSs, on adsorption of ssDNA on its surface. The sensor had a linear response over 0.1 to 100 ng/mL with a detection limit of 300 pg/mL and an excellent selectivity [168].

Malaria is a health concern in many parts of the world and is responsible for a considerable loss of lives. The protozoan Plasmodium parasite causes malaria, and its early detection remains a big challenge. Kenry et al. used single-layer MoS_2_ sheets for the detection of Plasmodium lactate dehydrogenase (pLDH) protein [169]. FL intensity of high-affinity malaria biomarker aptamer quenches almost 90% after adding MoS_2_ because of van der Waals interaction. The quantitative analysis shows a linear restoration of FL intensity upon serial addition of 0 to 62.5 mM pLDH protein with a detection limit of 550 pM, much lower than that of the clinically accepted safe limit of a few nM. To practically use the biosensor, several other bioanalytes ranging from insulin to globulin, were also tested for FL restoration. Results showed high selectivity towards pLDH protein and low detection limit.

#### 3.3.2. Detection of Bacteria and Virus

Zhang et al. studied the FL quenching of 6-carboxyfluorescein (FAM) dye-labeled ssDNA using MoS_2_, TaS_2,_ and TiS_2_. The adsorption of FAM dye-labeled ssDNA probe aptamer corresponding to the Influenza A virus onto the TMD surface quenches its FL intensity. Among the three TMD NSs, TaS_2_ has the highest quenching capability of 99% [170]. The systematic FL intensity restoration shows a linear response in the 0 to 20 nM concentration range. The TaS_2_-based sensor has the lowest detection limit of 0.05 nM compared to 0.1 nM for MoS_2_ and 0.2 nM for TiS_2_.

#### 3.3.3. Detection of DNAs and Other Biomolecules

**Figure 5:**
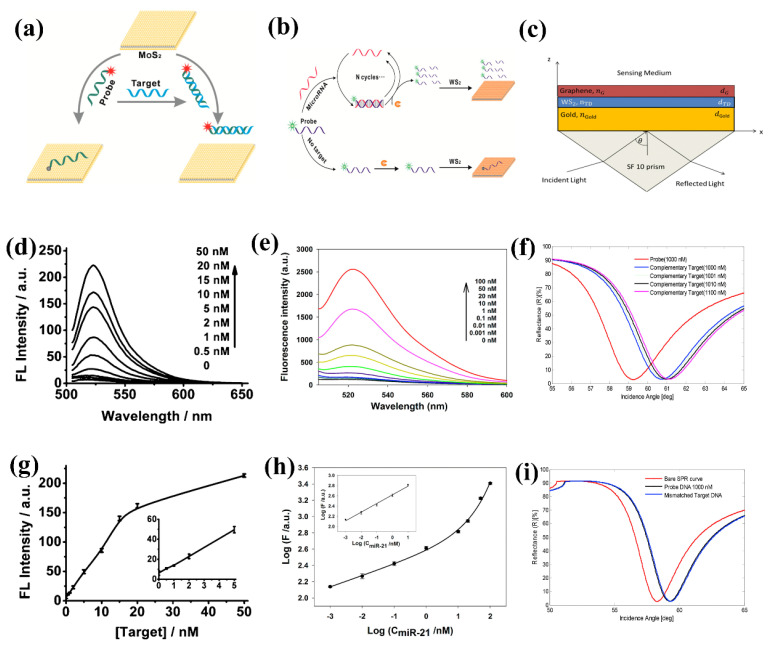
(**a**) DNA sensing using high fluorescence quenching properties of single-layer MoS_2_ NSs, Schematic representation; (**b**) Detection of miRNA using fluorescence quenching properties of WS_2_ NSs, Schematic of working principle; (**c**) Surface plasmon resonance-based DNA detector using graphene/WS_2_ hybrid structure, sensing platform schematic; (**d**) fluorescence intensity variation with DNA concentration; (**e**) response at different concentrations of miRNA; (**f**) response with targeted DNA; (**g**) Calibration curve for DNA sensing; (**h**) Calibration curve for miRNA detection; (**i**) response with non-targeted DNA. (**a**,**d**,**g**) reprinted with permission from ref. [66]. Copyrights 2013 American Chemical Society. (**b**,**e**,**h**) reprinted with permission from ref. [164]. Copyrights 2014 American Chemical Society. (**c**,**f**,**i**) reprinted with permission from ref. [171]. Copyright 2018 Elsevier.

An example of DNA sensing using fluorescence quenching [66,164,171] is shown in Figure 5. Zhu et al. first demonstrated the FL quenching of single-layer MoS_2_ by the FRET mechanism in the vicinity of a biomolecule. The probe ssDNA has a higher affinity toward single-layer MoS_2_ than dsDNA; hence, the FL intensity almost quenches completely on the adsorption of ssDNA due to nonradiative energy transfer among them. Upon binding with the targeted DNA, the probe DNA hybridizes to dsDNA and detaches from the MoS_2_ surface, restoring the FL intensity. The quantitative study between the addition of the targeted DNA and the FL intensity restoration showed a linear relationship from 0 to 50 nM concentration range with a detection limit of 500 pM [66]. Ge et al. designed FL based aptasensor for specific detection of adenosine triphosphate (ATP) and human α-thrombin in the human serum [172]. FL intensity of the probe aptamers diminished once MoS_2_ NSs were added because of the charge transfer. With the attachment of the targeted ATP and α-thrombin, the aptamers formed a dsDNA hybrid and detached from the MoS_2_ surface, resulting in FL restoration. However, even after the addition of a high concentration of targeted DNA, the FL intensity did not restore to its original value as some of the aptamers bound with MoS_2_ in other configurations and remained bound to the MoS_2_ NSs. The concentration-dependent study shows a linear response over a wide range of 0 to 2 mM for ATP. The system successfully detected ATP in human serum and from the extraction of lung adenocarcinoma A549 cells, proving its application in real-time PoC devices. A detection limit of 4 µM ATP and 300 pM of thrombin was reported using a similar mechanism.

Loan et al. made a graphene-encapsulated MoS_2_ heterostructure-based DNA sensor using a PL enhancement study. The ssDNA attachment acts as a positive voltage gating to graphene, changing the optoelectronic properties. The biocompatible top graphene layer protects the MoS_2_ surface from the aqueous medium. The functional groups on the graphene surface also serve as biolinkers between the aptamers and the graphene surface. The systematic enhancement of the PL intensity with the DNA concentration shows a linear behavior from 1 aM to 1 fM concentration. A similar study with single-sequence mismatch DNA shows that the enhancement is only due to targeted binding with the probe DNA, and the sensor has excellent selectivity toward the targeted DNA [173].

Huang et al. developed a microfluidic-based DNA sensor for rapid and multiple DNA screening using single-layer MoS_2_ NSs [174]. Polydimethylsiloxane (PDMS)-assisted zigzag-shaped microchannel helped the screening of multiple samples simultaneously and ensured uniform mixing of ssDNA and MoS_2_ NSs. Using the microchannel, rapid screening of DNA can detect targeted DNA with fM concentration within a few minutes. Wang and his co-workers used Peptide nucleic acid (PNA) as a probe for DNA sensing. PNAs are similar to ssDNA, with a higher binding affinity toward targeted DNA. Similar to ssDNA, the FL intensity of probe PNA quenches entirely after adsorbing on the WS_2_ surface because of charge transfer among them. The restoration of FL correlated with the binding of specified targeted DNA, which was confirmed by adding a single base mismatch DNA. The result showed almost negligible restoration of FL intensity, which indicated the precise binding nature of the PNAs [175]. The linear detection capability of the sensing device was in the range of 1 nM to 20 nM with a detection limit of 500 pM. Jin et al. functionalized a single-layer MoS_2_ surface with thiol-modified aptamer through SH–Au bond [46]. The thiol-Au bond enhanced the adsorption of ssDNA on the surface of a single-layer MoS_2_. The adsorption of ssDNA modulates the local dielectric constant and affects the band energy. There is a continuous blueshift with the addition of modified aptamers. The study reveals that one can detect targeted DNA at an nM concentration level by employing PL spectroscopy measurement on a single-layer MoS_2_. Xi et al. synthesized thioglycolic acid (TGA) functionalized single-layer MoS_2_ NSs to detect dopamine. Upon the addition of DA, the FL intensity of TGA functionalized MoS_2_ NSs quenches. In the presence of DA, the C–O⋯H–O hydrogen bonding starts stacking single-layer MoS_2_ NSs and results in FL quenching as a result of the charge transfers. Dopamine interacts with the TGA functionalized MoS_2_ by hydrogen bonding [176]. Gao et al. demonstrated thrombin detection using Au NPs modified MoS_2_ NSs. The FL-based biosensor had a linear responsivity from 50 nM to 20 µM concentration of DA with a detection limit of 2.7 nM [177].

Table 3 lists the optical biosensors discussed in this review. There are currently several optical-based biosensors commercially available for quantitative and qualitative analysis of bioanalytes. The commercial sensors include chemiluminescence assays, fluorometric assays, and various forms of ELISA kits. One of the challenges includes lengthy sample preparation steps and expensive kits [178,179]. As discussed earlier, the advantages of excellent optical properties, such as tunable bandgap, high FL emission, and quenching, provide TMDs with an excellent opportunity for future label-free sensing. In general, optical sensing systems are complex compared to electrical transduction-based systems, and TMD-based biosensing is no exception either.

## 4. Future Perspectives

There has been enormous research on 2D TMD materials since the discovery of graphene, but their application in the healthcare domain remains largely unexplored. State-of-the-art PoC products based on TMDs have not been commercialized. There is a need for robust study regarding wafer-scale synthesis, fabrication processes, and integration with state-of-the-art modern electronic fabrication. One of the critical challenges for TMDs to compete with existing technologies is to devise a low-cost wafer-scale synthesis solution. A uniform wafer-scale synthesis is an essential criterion for mass production; otherwise, the device-to-device variation could forestall the way for a PoC sensor to the consumers. Compared to 0D and 1D materials, the planner structure of 2D TMDs makes them inherently compatible with the existing state of art fabrication technologies for biosensors. Synthesis methods, such as CVD, MOCVD, and ALD, have the potential for high-quality synthesis and hence minimal device-to-device variation.

TMDs are suitable for new-generation wearable healthcare devices with their high mechanical flexibility and stability. They are generally grown at high temperatures, but flexible polymer substrates are incompatible with direct growth. TMDs are transferred from the growth substrate to the device substrate. Currently, ultrathin TMDs are transferred using Polymethyl methacrylate (PMMA) based on wet chemical methods [26]. The transfer samples contain PMMA residue, which creates defects and wrinkles. There is a big challenge for efficient wafer-scale transfer of 2D TMDs without creating defects or wrinkles. Thus, an improved transfer process is needed for highly efficient device fabrications or to find a solution for low-temperature growth directly on the flexible substrate. As part of the sensing application, functionalization of the TMD surface is essential for highly selective biosensors and minimizing false positive responses. There is a need for efficient surface engineering for the attachment of bioreceptor. Enormous work has been done during the last decade, and further improvements for suitable surface modification are still needed.

Once the scientific community resolves the present technological challenges, the 2D-TMD-based sensing platform has an excellent opportunity for next-generation personalized healthcare devices. With their mechanical flexibility, ultrathin thickness, and optical transparency, 2D-TMDs have the potential to incorporate into textiles for continuous monitoring of health conditions. Two-D TMDs have already demonstrated label-free detection of bioanalytes down to aM concentration, much lower than many current technologies, suggesting tremendous potential for next-generation personalized sensing platforms. The scientific community has made enormous progress in 2D TMD-based materials for biosensing applications in the last ten years. Further development and realizations are needed for sustainable academic research and collaboration with industrial partners to achieve next-generation applications in personalized health monitoring, wearable technologies, and low-power, portable diagnostics with superior performance compared to existing technologies.

## Figures and Tables

**Table 2:** Summary of TMD-based electrochemical biosensors.

Matrix	Method	Target Analyte	Linear Range	LoD	Reference
MWCNT/MoS_2_	CV	Dopamine	2150–5540 µM	13 nM	[135]
MoS_2_	ESI and DPV	miRNA-21	10 fM–1 nM	0.45 fM and 0.78 fM	[136]
MoS_2_	SWV	miRNA-21	1 pM–10 nM	0.26 pM	[61]
MoS_2_	SWV	miRNA	1 pM–1.5 nM	0.48 pM	[137]
MoS_2_-GO	CV	CEA	0.01 pg/mL–100 ng/mL	1.6 fg/mL	[140]
MoS_2_/T_3_C_4_	DPV	miRNA-182	1 fM–0.1 nM	0.43 fM	[141]
MoS_2_	CV	miRNA-155	1 fM–10 nM	0.32 fM	[143]
MoS_2_	Electrical	GSH	10 µM–500 mM	10 µM	[144]
MoS_2_@MWCNTs	EIS	HBeAg	0.1–500 pg/mL	26 fg/mL	[62]
MoS_2_	SWV	DNA	0.09–1.9 ng/mL	0.09 pg/mL	[146]
ZnO/MoS_2_	DPV	DNA	1 fM–1 µM	0.66 fM	[147]
MoS_2_	CV	DNA	0.1 fM–100 pM	0.018 fM	[148]
MoS_2_	DPV	Kanamycin	0.1–100 nM	0.03 nM	[149]
WS_2_/Graphite	CV	Adenine/Guanine	0.5–20 µM	50 nM and 90 nM	[150]
MoS_2_	CV	H_2_O_2_	5 nM–100 nM	2.5 nM	[153]
MoS_2_	Absorbance	H_2_O_2_	0.5–50 µM	0.5 µM	[154]
MoS_2_	CV	H_2_O_2_	0.23 µM–14.2 mM	0.2 µM	[155]
MoS_2_	CV	Glucose	10 µM–3 mM	1.08 µM	[161]

**Table 3:** Summary of TMD-based electrochemical biosensors.

Matrix	Method	Target Analyte	Linear Range	LoD	Reference
MoS_2_	FL	PSA	0.5–300 ng/mL	0.2 ng/ml	[162]
MoS_2_ and MoSe_2_	FL	PSA	0.2–100 nM	72 pM and 157 pM	[163]
WS_2_	FL	miRNA-21	1 pM–100 nM	300 fM	[164]
MoS_2_	FL	miRNA-21	0–40 nM	500 pM	[165]
MoS_2_	PL	miRNA-21			[166]
MoS_2_	SPR	CYFRA21-1	0.05 pg/mL–100 ng/mL	0.05 pg/ml	[63]
MoS_2_	FL	CEA	0.1–100 ng/mL	300 pg/ml	[168]
MoS_2_	FL	pLDH	0–62.5 mM	550 pM	[169]
MoS_2_, TaS_2_, and TiS_2_	FL	DNA	0–20 nM	0.1 nM, 0.05 nM and 0.2 nM	[170]
MoS_2_	FL	DNA	0–50 nM	500 pM	[66]
MoS_2_	FL	ATP and α-Thrombin	0–2 mM for ATP	4 µM and 300 pM	[172]
Graphene/MoS_2_	PL	DNA	1 aM–1 fM	1 aM	[173]
MoS_2_	FL	DNA	0–20 nM	500 pM	[174]
WS_2_	FL	DNA	1–20 nM	500 pM	[175]
MoS_2_	PL	DNA	1 nM–20 µM	1 nM	[46]
MoS_2_	FL	DA	0.05–20 µM	27 nM	[176]
MoS_2_	FL	Thrombin	500 fM–20 nM	6 fM	[177]

## Data Availability

No new data was generated, as this is a review paper.

## References

[1] Novoselov K.S., Geim A.K., Morozov S.V., Jiang D., Zhang Y., Dubonos S.V., Grigorieva I.V., Firsov A.A. (2004). Electric Field Effect in Atomically Thin Carbon Films. Science.

[2] Bolotin K.I., Sikes K.J., Jiang Z., Klima M., Fudenberg G., Hone J., Kim P., Stormer H.L. (2008). Ultrahigh Electron Mobility in Suspended Graphene. Solid State Commun..

[3] Xia F., Mueller T., Lin Y., Valdes-Garcia A., Avouris P. (2009). Ultrafast Graphene Photodetector. Nat. Nanotechol..

[4] Lin Y.-M., Jenkins K.A., Valdes-Garcia A., Small J.P., Farmer D.B., Avouris P. (2009). Operation of Graphene Transistors at Gigahertz Frequencies. Nano Lett..

[5] Mittendorff M., Winnerl S., Kamann J., Eroms J., Weiss D., Schneider H., Helm M. (2013). Ultrafast Graphene-Based Broadband THz Detector. Appl. Phys. Lett..

[6] Mueller T., Xia F., Avouris P. (2010). Graphene Photodetectors for High-Speed Optical Communications. Nat. Photon..

[7] Zhang B.Y., Liu T., Meng B., Li X., Liang G., Hu X., Wang Q.J. (2013). Broadband High Photoresponse from Pure Monolayer Graphene Photodetector. Nat. Commun..

[8] Yoon H.J., Jun D.H., Yang J.H., Zhou Z., Yang S.S., Cheng M.M.-C. (2011). Carbon Dioxide Gas Sensor Using a Graphene Sheet. Sens. Actuators B Chem..

[9] Tian W., Liu X., Yu W. (2018). Research Progress of Gas Sensor Based on Graphene and Its Derivatives: A Review. Appl. Sci..

[10] Justino C.I.L., Gomes A.R., Freitas A.C., Duarte A.C., Rocha-Santos T.A.P. (2017). Graphene Based Sensors and Biosensors. TrAC Trends Anal. Chem..

[11] De S., Coleman J.N. (2010). Are There Fundamental Limitations on the Sheet Resistance and Transmittance of Thin Graphene Films?. ACS Nano.

[12] Novoselov K.S., Geim A.K., Morozov S.V., Jiang D., Katsnelson M.I., Grigorieva I.V., Dubonos S.V., Firsov A.A. (2005). Two-Dimensional Gas of Massless Dirac Fermions in Graphene. Nature.

[13] Lee S.Y., Duong D.L., Vu Q.A., Jin Y., Kim P., Lee Y.H. (2015). Chemically Modulated Band Gap in Bilayer Graphene Memory Transistors with High On/Off Ratio. ACS Nano.

[14] Xiao S., Chen J.-H., Adam S., Williams E.D., Fuhrer M.S. (2010). Charged Impurity Scattering in Bilayer Graphene. Phys. Rev. B.

[15] Huu H.T., Thi X.D.N., Van K.N., Kim S.J., Vo V. (2019). A Facile Synthesis of MoS_2_/g-C_3_N_4_ Composite as an Anode Material with Improved Lithium Storage Capacity. Materials.

[16] Ping J., Fan Z., Sindoro M., Ying Y., Zhang H. (2017). Recent Advances in Sensing Applications of Two-Dimensional Transition Metal Dichalcogenide Nanosheets and Their Composites. Adv. Funct. Mater..

[17] Molaei M.J. (2021). Two-Dimensional (2D) Materials beyond Graphene in Cancer Drug Delivery, Photothermal and Photodynamic Therapy, Recent Advances and Challenges Ahead: A Review. J. Drug Deliv. Sci. Technol..

[18] Bolotsky A., Butler D., Dong C., Gerace K., Glavin N.R., Muratore C., Robinson J.A., Ebrahimi A. (2019). Two-Dimensional Materials in Biosensing and Healthcare: From In Vitro Diagnostics to Optogenetics and Beyond. ACS Nano.

[19] Sinha A., Dhanjai, Zhao H., Huang Y., Lu X., Chen J., Jain R. (2018). MXene: An Emerging Material for Sensing and Biosensing. TrAC Trends Anal. Chem..

[20] Ménard-Moyon C., Bianco A., Kalantar-Zadeh K. (2020). Two-Dimensional Material-Based Biosensors for Virus Detection. ACS Sens..

[21] Garg M., Gupta A., Sharma A.L., Singh S. (2021). Advancements in 2D Materials Based Biosensors for Oxidative Stress Biomarkers. ACS Appl. Bio Mater..

[22] Zheng W., Jiang Y., Hu X., Li H., Zeng Z., Wang X., Pan A. (2018). Light Emission Properties of 2D Transition Metal Dichalcogenides: Fundamentals and Applications. Adv. Opt. Mater..

[23] Choi W., Choudhary N., Han G.H., Park J., Akinwande D., Lee Y.H. (2017). Recent Development of Two-Dimensional Transition Metal Dichalcogenides and Their Applications. Mater. Today.

[24] Lv R., Robinson J.A., Schaak R.E., Sun D., Sun Y., Mallouk T.E., Terrones M. (2015). Transition Metal Dichalcogenides and Beyond: Synthesis, Properties, and Applications of Single- and Few-Layer Nanosheets. Acc. Chem. Res..

[25] Sebastian A., Pendurthi R., Choudhury T.H., Redwing J.M., Das S. (2021). Benchmarking Monolayer MoS_2_ and WS_2_ Field-Effect Transistors. Nat. Commun..

[26] Das S., Sebastian A., Pop E., McClellan C.J., Franklin A.D., Grasser T., Knobloch T., Illarionov Y., Penumatcha A.V., Appenzeller J. (2021). Transistors Based on Two-Dimensional Materials for Future Integrated Circuits. Nat. Electron..

[27] Mawlong L.P., Paul K.K., Giri P.K. (2019). Simultaneous Photoluminescence Enhancement in CVD Grown Single Layer MoS_2_ and TiO_2_ NRs in the MoS_2_@TiO_2_ Heterojunction. Proceedings of the AIP Conference Proceedings.

[28] Zhu B., Chen X., Cui X. (2015). Exciton Binding Energy of Monolayer WS_2_. Sci. Rep..

[29] Mei J., Li Y.-T., Zhang H., Xiao M.-M., Ning Y., Zhang Z.-Y., Zhang G.-J. (2018). Molybdenum Disulfide Field-Effect Transistor Biosensor for Ultrasensitive Detection of DNA by Employing Morpholino as Probe. Biosens. Bioelectron..

[30] Chen J., Wang Q., Sheng Y., Cao G., Yang P., Shan Y., Liao F., Muhammad Z., Bao W., Hu L. (2019). High-Performance WSe_2_ Photodetector Based on a Laser-Induced p–n Junction. ACS Appl. Mater. Interfaces.

[31] Liu T., Wang C., Gu X., Gong H., Cheng L., Shi X., Feng L., Sun B., Liu Z. (2014). Drug Delivery with PEGylated MoS_2_ Nano-Sheets for Combined Photothermal and Chemotherapy of Cancer. Adv. Mater..

[32] Li B.L., Setyawati M.I., Chen L., Xie J., Ariga K., Lim C.-T., Garaj S., Leong D.T. (2017). Directing Assembly and Disassembly of 2D MoS_2_ Nanosheets with DNA for Drug Delivery. ACS Appl. Mater. Interfaces.

[33] Pumera M., Loo A.H. (2014). Layered Transition-Metal Dichalcogenides (MoS_2_ and WS_2_) for Sensing and Biosensing. TrAC Trends Anal. Chem..

[34] Kim S.-G., Kim S.-H., Park J., Kim G.-S., Park J.-H., Saraswat K.C., Kim J., Yu H.-Y. (2019). Infrared Detectable MoS_2_ Phototransistor and Its Application to Artificial Multilevel Optic-Neural Synapse. ACS Nano.

[35] Li X., Li X., Li Z., Wang J., Zhang J. (2017). WS_2_ Nanoflakes Based Selective Ammonia Sensors at Room Temperature. Sens. Actuators B Chem..

[36] Liu J., Chen X., Wang Q., Xiao M., Zhong D., Sun W., Zhang G., Zhang Z. (2019). Ultrasensitive Monolayer MoS_2_ Field-Effect Transistor Based DNA Sensors for Screening of Down Syndrome. Nano Lett..

[37] Gutiérrez H.R., Perea-López N., Elías A.L., Berkdemir A., Wang B., Lv R., López-Urías F., Crespi V.H., Terrones H., Terrones M. (2013). Extraordinary Room-Temperature Photoluminescence in Triangular WS_2_ Monolayers. Nano Lett..

[38] Kang M., Kim B., Ryu S.H., Jung S.W., Kim J., Moreschini L., Jozwiak C., Rotenberg E., Bostwick A., Kim K.S. (2017). Universal Mechanism of Band-Gap Engineering in Transition-Metal Dichalcogenides. Nano Lett..

[39] Kang K., Xie S., Huang L., Han Y., Huang P.Y., Mak K.F., Kim C.-J., Muller D., Park J. (2015). High-Mobility Three-Atom-Thick Semiconducting Films with Wafer-Scale Homogeneity. Nature.

[40] Lan C., Zhou Z., Zhou Z., Li C., Shu L., Shen L., Li D., Dong R., Yip S., Ho J.C. (2018). Wafer-Scale Synthesis of Monolayer WS_2_ for High-Performance Flexible Photodetectors by Enhanced Chemical Vapor Deposition. Nano Res..

[41] Bora A., Mawlong L.P., Giri P.K. (2021). Highly Suppressed Dark Current and Fast Photoresponse from Au Nanoparticle-Embedded, Si/Au/WS_2_ Quantum-Dot-Based, Self-Biased Schottky Photodetectors. ACS Appl. Electron. Mater..

[42] Ovchinnikov D., Allain A., Huang Y.-S., Dumcenco D., Kis A. (2014). Electrical Transport Properties of Single-Layer WS_2_. ACS Nano.

[43] Huiling Loo A., Bonanni A., Pumera M. (2016). Strong Dependence of Fluorescence Quenching on the Transition Metal in Layered Transition Metal Dichalcogenide Nanoflakes for Nucleic Acid Detection. Analyst.

[44] Kim J., Seung H., Kang D., Kim J., Bae H., Park H., Kang S., Choi C., Choi B.K., Kim J.S. (2021). Wafer-Scale Production of Transition Metal Dichalcogenides and Alloy Monolayers by Nanocrystal Conversion for Large-Scale Ultrathin Flexible Electronics. Nano Lett..

[45] Pereira N.M., Rezende N.P., Cunha T.H.R., Barboza A.P.M., Silva G.G., Lippross D., Neves B.R.A., Chacham H., Ferlauto A.S., Lacerda R.G. (2022). Aerosol-Printed MoS_2_ Ink as a High Sensitivity Humidity Sensor. ACS Omega.

[46] Jin K., Xie L., Tian Y., Liu D. (2016). Au-Modified Monolayer MoS_2_ Sensor for DNA Detection. J. Phys. Chem. C.

[47] KiKrubasankar B., Won Y.S., Adofo L.A., Choi S.H., Kim S.M., Kim K.K. (2022). Atomic and Structural Modifications of Two-Dimensional Transition Metal Dichalcogenides for Various Advanced Applications. Chem. Sci..

[48] Nolan M., O’Callaghan S., Fagas G., Greer J.C., Frauenheim T. (2007). Silicon Nanowire Band Gap Modification. Nano Lett..

[49] Xu M., Liang T., Shi M., Chen H. (2013). Graphene-Like Two-Dimensional Materials. Chem. Rev..

[50] Rao R., Pint C.L., Islam A.E., Weatherup R.S., Hofmann S., Meshot E.R., Wu F., Zhou C., Dee N., Amama P.B. (2018). Carbon Nanotubes and Related Nanomaterials: Critical Advances and Challenges for Synthesis toward Mainstream Commercial Applications. ACS Nano.

[51] George A.S., Mutlu Z., Ionescu R., Wu R.J., Jeong J.S., Bay H.H., Chai Y., Mkhoyan K.A., Ozkan M., Ozkan C.S. (2014). Wafer Scale Synthesis and High Resolution Structural Characterization of Atomically Thin MoS_2_ Layers. Adv. Funct. Mater..

[52] Chubarov M., Choudhury T.H., Hickey D.R., Bachu S., Zhang T., Sebastian A., Bansal A., Zhu H., Trainor N., Das S. (2021). Wafer-Scale Epitaxial Growth of Unidirectional WS_2_ Monolayers on Sapphire. ACS Nano.

[53] He Q., Zeng Z., Yin Z., Li H., Wu S., Huang X., Zhang H. (2012). Fabrication of Flexible MoS_2_ Thin-Film Transistor Arrays for Practical Gas-Sensing Applications. Small.

[54] Kong L., Li G., Su Q., Zhang X., Liu Z., Liao G., Sun B., Shi T. (2022). Inkjet-Printed, Large-Area, Flexible Photodetector Array Based on Electrochemical Exfoliated MoS_2_ Film for Photoimaging. Adv. Eng. Mater..

[55] Quereda J., Kuriakose S., Munuera C., Mompean F.J., Al-Enizi A.M., Nafady A., Diez E., Frisenda R., Castellanos-Gomez A. (2022). Scalable and Low-Cost Fabrication of Flexible WS_2_ Photodetectors on Polycarbonate. npj Flex. Electron..

[56] Majd S.M., Salimi A., Ghasemi F. (2018). An Ultrasensitive Detection of MiRNA-155 in Breast Cancer via Direct Hybridization Assay Using Two-Dimensional Molybdenum Disulfide Field-Effect Transistor Biosensor. Biosens. Bioelectron..

[57] Lee J., Dak P., Lee Y., Park H., Choi W., Alam M.A., Kim S. (2014). Two-Dimensional Layered MoS_2_ Biosensors Enable Highly Sensitive Detection of Biomolecules. Sci. Rep..

[58] Liu G., Robertson A.W., Li M.M.-J., Kuo W.C.H., Darby M.T., Muhieddine M.H., Lin Y.-C., Suenaga K., Stamatakis M., Warner J.H. (2017). MoS_2_ Monolayer Catalyst Doped with Isolated Co Atoms for the Hydrodeoxygenation Reaction. Nat. Chem..

[59] Sarkar D., Liu W., Xie X., Anselmo A.C., Mitragotri S., Banerjee K. (2014). MoS_2_ Field-Effect Transistor for Next-Generation Label-Free Biosensors. ACS Nano.

[60] Hossain M.M., Shabbir B., Wu Y., Yu W., Krishnamurthi V., Uddin H., Mahmood N., Walia S., Bao Q., Alan T. (2021). Ultrasensitive WSe2 Field-Effect Transistor-Based Biosensor for Label-Free Detection of Cancer in Point-of-Care Applications. 2D Mater..

[61] Zhu D., Liu W., Zhao D., Hao Q., Li J., Huang J., Shi J., Chao J., Su S., Wang L. (2017). Label-Free Electrochemical Sensing Platform for MicroRNA-21 Detection Using Thionine and Gold Nanoparticles Co-Functionalized MoS_2_ Nanosheet. ACS Appl. Mater. Interfaces.

[62] Gao Z., Li Y., Zhang X., Feng J., Kong L., Wang P., Chen Z., Dong Y., Wei Q. (2018). Ultrasensitive Electrochemical Immunosensor for Quantitative Detection of HBeAg Using Au@Pd/MoS_2_@MWCNTs Nanocomposite as Enzyme-Mimetic Labels. Biosens. Bioelectron..

[63] Chiu N.-F., Yang H.-T. (2020). High-Sensitivity Detection of the Lung Cancer Biomarker CYFRA21-1 in Serum Samples Using a Carboxyl-MoS_2_ Functional Film for SPR-Based Immunosensors. Front. Bioeng. Biotechnol..

[64] Liu K., Zhang J., Jiang J., Xu T., Wang S., Chang P., Zhang Z., Ma J., Liu T. (2020). MoSe2-Au Based Sensitivity Enhanced Optical Fiber Surface Plasmon Resonance Biosensor for Detection of Goat-Anti-Rabbit IgG. IEEE Access.

[65] Qiu X., Hildebrandt N. (2015). Rapid and Multiplexed MicroRNA Diagnostic Assay Using Quantum Dot-Based Förster Resonance Energy Transfer. ACS Nano.

[66] Zhu C., Zeng Z., Li H., Li F., Fan C., Zhang H. (2013). Single-Layer MoS_2_-Based Nanoprobes for Homogeneous Detection of Biomolecules. J. Am. Chem. Soc..

[67] Singh P., Gupta R., Sinha M., Kumar R., Bhalla V. (2016). MoS_2_ Based Digital Response Platform for Aptamer Based Fluorescent Detection of Pathogens. Microchim. Acta.

[68] Sun X., Fan J., Fu C., Yao L., Zhao S., Wang J., Xiao J. (2017). WS_2_ and MoS_2_ Biosensing Platforms Using Peptides as Probe Biomolecules. Sci. Rep..

[69] Oudeng G., Au M., Shi J., Wen C., Yang M. (2018). One-Step in Situ Detection of MiRNA-21 Expression in Single Cancer Cells Based on Biofunctionalized MoS_2_ Nanosheets. ACS Appl. Mater. Interfaces.

[70] Park H., Baek S., Sen A., Jung B., Shim J., Park Y.C., Lee L.P., Kim Y.J., Kim S. (2022). Ultrasensitive and Selective Field-Effect Transistor-Based Biosensor Created by Rings of MoS_2_ Nanopores. ACS Nano.

[71] Li H., Wu J., Yin Z., Zhang H. (2014). Preparation and Applications of Mechanically Exfoliated Single-Layer and Multilayer MoS_2_ and WSe2 Nanosheets. Acc. Chem. Res..

[72] Paradisanos I., Germanis S., Pelekanos N.T., Fotakis C., Kymakis E., Kioseoglou G., Stratakis E. (2017). Room Temperature Observation of Biexcitons in Exfoliated WS_2_ Monolayers. Appl. Phys. Lett..

[73] Budania P., Baine P.T., Montgomery J.H., McNeill D.W., Neil Mitchell S.J., Modreanu M., Hurley P.K. (2017). Comparison between Scotch Tape and Gel-Assisted Mechanical Exfoliation Techniques for Preparation of 2D Transition Metal Dichalcogenide Flakes. Micro Nano Lett..

[74] Cui Q., Luo Z., Cui Q., Zhu W., Shou H., Wu C., Liu Z., Lin Y., Zhang P., Wei S. (2021). Robust and High Photoluminescence in WS_2_ Monolayer through In Situ Defect Engineering. Adv. Funct. Mater..

[75] Plechinger G., Nagler P., Kraus J., Paradiso N., Strunk C., Schüller C., Korn T. (2015). Identification of Excitons, Trions and Biexcitons in Single-Layer WS_2_. Phys. Status Solidi (RRL)—Rapid Res. Lett..

[76] Larentis S., Fallahazad B., Tutuc E. (2012). Field-Effect Transistors and Intrinsic Mobility in Ultra-Thin MoSe_2_ Layers. Appl. Phys. Lett..

[77] Shim G.W., Yoo K., Seo S.-B., Shin J., Jung D.Y., Kang I.-S., Ahn C.W., Cho B.J., Choi S.-Y. (2014). Large-Area Single-Layer MoSe_2_ and Its van Der Waals Heterostructures. ACS Nano.

[78] Li H., Lu G., Wang Y., Yin Z., Cong C., He Q., Wang L., Ding F., Yu T., Zhang H. (2013). Mechanical Exfoliation and Characterization of Single- and Few-Layer Nanosheets of WSe_2_, TaS_2_, and TaSe_2_. Small.

[79] Wang L., Wang Y., Wong J.I., Palacios T., Kong J., Yang H.Y. (2014). Functionalized MoS_2_ Nanosheet-Based Field-Effect Biosensor for Label-Free Sensitive Detection of Cancer Marker Proteins in Solution. Small.

[80] Özgür D.Ö., Özkan G., Atakol O., Çelikkan H. (2021). Facile Ion-Exchange Method for Zn Intercalated MoS_2_ As an Efficient and Stable Catalyst toward Hydrogen Evaluation Reaction. ACS Appl. Energy Mater..

[81] Liu H., Chen X., Deng L., Ding M., Li J., He X. (2016). Perpendicular Growth of Few-Layered MoS_2_ Nanosheets on MoO_3_ Nanowires Fabricated by Direct Anion Exchange Reactions for High-Performance Lithium-Ion Batteries. J. Mater. Chem. A.

[82] Anto Jeffery A., Nethravathi C., Rajamathi M. (2014). Two-Dimensional Nanosheets and Layered Hybrids of MoS_2_ and WS_2_ through Exfoliation of Ammoniated MS2 (M = Mo, W). J. Phys. Chem. C.

[83] Zhu X., Su Z., Wu C., Cong H., Ai X., Yang H., Qian J. (2022). Exfoliation of MoS_2_ Nanosheets Enabled by a Redox-Potential-Matched Chemical Lithiation Reaction. Nano Lett..

[84] Patel A.B., Machhi H.K., Chauhan P., Narayan S., Dixit V., Soni S.S., Jha P.K., Solanki G.K., Patel K.D., Pathak V.M. (2019). Electrophoretically Deposited MoSe2/WSe2 Heterojunction from Ultrasonically Exfoliated Nanocrystals for Enhanced Electrochemical Photoresponse. ACS Appl. Mater. Interfaces.

[85] Hussain S., Singh J., Vikraman D., Singh A.K., Iqbal M.Z., Khan M.F., Kumar P., Choi D.-C., Song W., An K.-S. (2016). Large-Area, Continuous and High Electrical Performances of Bilayer to Few Layers MoS_2_ Fabricated by RF Sputtering via Post-Deposition Annealing Method. Sci. Rep..

[86] Akhtaruzzaman M., Shahiduzzaman M., Amin N., Muhammad G., Islam M.A., Rafiq K.S.B., Sopian K. (2021). Impact of Ar Flow Rates on Micro-Structural Properties of WS_2_ Thin Film by RF Magnetron Sputtering. Nanomaterials.

[87] Chaudhary N., Khanuja M., Abid, Islam S.S. (2018). Hydrothermal Synthesis of MoS_2_ Nanosheets for Multiple Wavelength Optical Sensing Applications. Sens. Actuators A Phys..

[88] Wan X., Chen K., Chen Z., Xie F., Zeng X., Xie W., Chen J., Xu J. (2017). Controlled Electrochemical Deposition of Large-Area MoS_2_ on Graphene for High-Responsivity Photodetectors. Adv. Funct. Mater..

[89] Cong C., Shang J., Wu X., Cao B., Peimyoo N., Qiu C., Sun L., Yu T. (2014). Synthesis and Optical Properties of Large-Area Single-Crystalline 2D Semiconductor WS_2_ Monolayer from Chemical Vapor Deposition. Adv. Opt. Mater..

[90] Huang K.-J., Zhang J.-Z., Shi G.-W., Liu Y.-M. (2014). Hydrothermal Synthesis of Molybdenum Disulfide Nanosheets as Supercapacitors Electrode Material. Electrochim. Acta.

[91] Xu T., Liu Y., Pei Y., Chen Y., Jiang Z., Shi Z., Xu J., Wu D., Tian Y., Li X. (2018). The Ultra-High NO2 Response of Ultra-Thin WS_2_ Nanosheets Synthesized by Hydrothermal and Calcination Processes. Sens. Actuators B Chem..

[92] Chen Y., Pei Y., Jiang Z., Shi Z., Xu J., Wu D., Xu T., Tian Y., Wang X., Li X. (2018). Humidity Sensing Properties of the Hydrothermally Synthesized WS_2_-Modified SnO2 Hybrid Nanocomposite. Appl. Surf. Sci..

[93] Zhang D., Liu T., Cheng J., Cao Q., Zheng G., Liang S., Wang H., Cao M.-S. (2019). Lightweight and High-Performance Microwave Absorber Based on 2D WS_2_–RGO Heterostructures. Nano-Micro Lett..

[94] Sakthivel R., Keerthi M., Chung R.-J., He J.-H. (2023). Heterostructures of 2D Materials and Their Applications in Biosensing. Prog. Mater. Sci..

[95] Noori Y.J., Thomas S., Ramadan S., Smith D.E., Greenacre V.K., Abdelazim N., Han Y., Beanland R., Hector A.L., Klein N. (2020). Large-Area Electrodeposition of Few-Layer MoS_2_ on Graphene for 2D Material Heterostructures. ACS Appl. Mater. Interfaces.

[96] Thomas S., Greenacre V.K., Smith D.E., Noori Y.J., Abdelazim N.M., Hector A.L., de Groot C.H., Levason W., Bartlett P.N., Reid G. (2021). Tungsten Disulfide Thin Films via Electrodeposition from a Single Source Precursor. Chem. Commun..

[97] Zazpe R., Charvot J., Krumpolec R., Hromádko L., Pavliňák D., Dvorak F., Knotek P., Michalicka J., Přikryl J., Ng S. (2020). Atomic Layer Deposition of MoSe2 Using New Selenium Precursors. FlatChem.

[98] Zazpe R., Krumpolec R., Sopha H., Rodriguez-Pereira J., Charvot J., Hromádko L., Kolíbalová E., Michalička J., Pavliňák D., Motola M. (2020). Atomic Layer Deposition of MoSe_2_ Nanosheets on TiO_2_ Nanotube Arrays for Photocatalytic Dye Degradation and Electrocatalytic Hydrogen Evolution. ACS Appl. Nano Mater..

[99] Wu Y., Raza M.H., Chen Y.-C., Amsalem P., Wahl S., Skrodczky K., Xu X., Lokare K.S., Zhukush M., Gaval P. (2019). A Self-Limited Atomic Layer Deposition of WS_2_ Based on the Chemisorption and Reduction of Bis(t-Butylimino)Bis(Dimethylamino) Complexes. Chem. Mater..

[100] Sperling B.A., Kalanyan B., Maslar J.E. (2020). Atomic Layer Deposition of Al_2_O_3_ Using Trimethylaluminum and H_2_O: The Kinetics of the H_2_O Half-Cycle. J. Phys. Chem. C.

[101] Tai T.B., Cao L., Mattelaer F., Rampelberg G., Hashemi F.S.M., Dendooven J., van Ommen J.R., Detavernier C., Reyniers M.-F. (2019). Atomic Layer Deposition of Al2O3 Using Aluminum Triisopropoxide (ATIP): A Combined Experimental and Theoretical Study. J. Phys. Chem. C.

[102] Liu H.F., Wong S.L., Chi D.Z. (2015). CVD Growth of MoS_2_-Based Two-Dimensional Materials. Chem. Vap. Depos..

[103] Zhan Y., Liu Z., Najmaei S., Ajayan P.M., Lou J. (2012). Large-Area Vapor-Phase Growth and Characterization of MoS_2_ Atomic Layers on a SiO_2_ Substrate. Small.

[104] Tao L., Chen K., Chen Z., Chen W., Gui X., Chen H., Li X., Xu J.-B. (2017). Centimeter-Scale CVD Growth of Highly Crystalline Single-Layer MoS_2_ Film with Spatial Homogeneity and the Visualization of Grain Boundaries. ACS Appl. Mater. Interfaces.

[105] Yorulmaz B., Özden A., Şar H., Ay F., Sevik C., Perkgöz N.K. (2019). CVD Growth of Monolayer WS_2_ through Controlled Seed Formation and Vapor Density. Mater. Sci. Semicond. Process..

[106] Zhang Z., Chen P., Yang X., Liu Y., Ma H., Li J., Zhao B., Luo J., Duan X., Duan X. (2020). Ultrafast Growth of Large Single Crystals of Monolayer WS_2_ and WSe_2_. Natl. Sci. Rev..

[107] Chen T., Sheng Y., Zhou Y., Chang R., Wang X., Huang H., Zhang Q., Hou L., Warner J.H. (2019). High Photoresponsivity in Ultrathin 2D Lateral Graphene:WS_2_:Graphene Photodetectors Using Direct CVD Growth. ACS Appl. Mater. Interfaces.

[108] Qi Z., Zhai X., Jiang X., Xu X., Fan C., Shen L., Xiao Q., Jiang S., Deng Q., Liu H. (2022). Epitaxy of NiTe2 on WS_2_ for the P-Type Schottky Contact and Increased Photoresponse. ACS Appl. Mater. Interfaces.

[109] Arnold A.J., Razavieh A., Nasr J.R., Schulman D.S., Eichfeld C.M., Das S. (2017). Mimicking Neurotransmitter Release in Chemical Synapses via Hysteresis Engineering in MoS_2_ Transistors. ACS Nano.

[110] Cohen A., Patsha A., Mohapatra P.K., Kazes M., Ranganathan K., Houben L., Oron D., Ismach A. (2021). Growth-Etch Metal–Organic Chemical Vapor Deposition Approach of WS_2_ Atomic Layers. ACS Nano.

[111] Cwik S., Mitoraj D., Mendoza Reyes O., Rogalla D., Peeters D., Kim J., Schütz H.M., Bock C., Beranek R., Devi A. (2018). Direct Growth of MoS_2_ and WS_2_ Layers by Metal Organic Chemical Vapor Deposition. Adv. Mater. Interfaces.

[112] Li J., Naiini M.M., Vaziri S., Lemme M.C., Östling M. (2014). Inkjet Printing of MoS_2_. Adv. Funct. Mater..

[113] Strimbu K., Tavel J.A. (2010). What Are Biomarkers?. Curr. Opin. HIV AIDS.

[114] Bray F., Ferlay J., Soerjomataram I., Siegel R.L., Torre L.A., Jemal A. (2018). Global Cancer Statistics 2018: GLOBOCAN Estimates of Incidence and Mortality Worldwide for 36 Cancers in 185 Countries. CA A Cancer J. Clin..

[115] Fathi-Hafshejani P., Azam N., Wang L., Kuroda M.A., Hamilton M.C., Hasim S., Mahjouri-Samani M. (2021). Two-Dimensional-Material-Based Field-Effect Transistor Biosensor for Detecting COVID-19 Virus (SARS-CoV-2). ACS Nano.

[116] Park H., Han G., Lee S.W., Lee H., Jeong S.H., Naqi M., AlMutairi A., Kim Y.J., Lee J., Kim W. (2017). Label-Free and Recalibrated Multilayer MoS_2_ Biosensor for Point-of-Care Diagnostics. ACS Appl. Mater. Interfaces.

[117] Park H., Lee H., Jeong S.H., Lee E., Lee W., Liu N., Yoon D.S., Kim S., Lee S.W. (2019). MoS_2_ Field-Effect Transistor-Amyloid-Β1–42 Hybrid Device for Signal Amplified Detection of MMP-9. Anal. Chem..

[118] Ilic D., Djulbegovic M., Jung J.H., Hwang E.C., Zhou Q., Cleves A., Agoritsas T., Dahm P. (2018). Prostate Cancer Screening with Prostate-Specific Antigen (PSA) Test: A Systematic Review and Meta-Analysis. BMJ.

[119] Yoo G., Park H., Kim M., Song W.G., Jeong S., Kim M.H., Lee H., Lee S.W., Hong Y.K., Lee M.G. (2017). Real-Time Electrical Detection of Epidermal Skin MoS_2_ Biosensor for Point-of-Care Diagnostics. Nano Res..

[120] Peng Y., Croce C.M. (2016). The Role of MicroRNAs in Human Cancer. Signal Transduct. Target. Ther..

[121] Zhang Y.-J., Li S., Gan R.-Y., Zhou T., Xu D.-P., Li H.-B. (2015). Impacts of Gut Bacteria on Human Health and Diseases. Int. J. Mol. Sci..

[122] Qureshi A., Niazi J.H. (2021). Biosensors for Detecting Viral and Bacterial Infections Using Host Biomarkers: A Review. Analyst.

[123] Thakur B., Zhou G., Chang J., Pu H., Jin B., Sui X., Yuan X., Yang C.-H., Magruder M., Chen J. (2018). Rapid Detection of Single E. Coli Bacteria Using a Graphene-Based Field-Effect Transistor Device. Biosens. Bioelectron..

[124] Kumar N., Wang W., Ortiz-Marquez J.C., Catalano M., Gray M., Biglari N., Hikari K., Ling X., Gao J., van Opijnen T. (2020). Dielectrophoresis Assisted Rapid, Selective and Single Cell Detection of Antibiotic Resistant Bacteria with G-FETs. Biosens. Bioelectron..

[125] Tan X., Yang M., Zhu L., Gunathilaka G., Zhou Z., Chen P.-Y., Zhang Y., Cheng M.M.-C. (2022). Ultrasensitive and Selective Bacteria Sensors Based on Functionalized Graphene Transistors. IEEE Sens. J..

[126] Sadighbayan D., Hasanzadeh M., Ghafar-Zadeh E. (2020). Biosensing Based on Field-Effect Transistors (FET): Recent Progress and Challenges. TrAC Trends Anal. Chem..

[127] Sengupta J., Hussain C.M. (2021). Graphene-Based Field-Effect Transistor Biosensors for the Rapid Detection and Analysis of Viruses: A Perspective in View of COVID-19. Carbon Trends.

[128] Moudgil A., Singh S., Mishra N., Mishra P., Das S. (2020). MoS_2_/TiO_2_ Hybrid Nanostructure-Based Field-Effect Transistor for Highly Sensitive, Selective, and Rapid Detection of Gram-Positive Bacteria. Adv. Mater. Technol..

[129] Lee D.-W., Lee J., Sohn I.Y., Kim B.-Y., Son Y.M., Bark H., Jung J., Choi M., Kim T.H., Lee C. (2015). Field-Effect Transistor with a Chemically Synthesized MoS_2_ Sensing Channel for Label-Free and Highly Sensitive Electrical Detection of DNA Hybridization. Nano Res..

[130] Bahri M., Shi B., Elaguech M.A., Djebbi K., Zhou D., Liang L., Tlili C., Wang D. (2022). Tungsten Disulfide Nanosheet-Based Field-Effect Transistor Biosensor for DNA Hybridization Detection. ACS Appl. Nano Mater..

[131] Nam H., Oh B.-R., Chen P., Chen M., Wi S., Wan W., Kurabayashi K., Liang X. (2015). Multiple MoS_2_ Transistors for Sensing Molecule Interaction Kinetics. Sci. Rep..

[132] Nam H., Oh B.-R., Chen M., Wi S., Li D., Kurabayashi K., Liang X. (2015). Fabrication and Comparison of MoS_2_ and WSe_2_ Field-Effect Transistor Biosensors. J. Vac. Sci. Technol. B.

[133] Chen X., Hao S., Zong B., Liu C., Mao S. (2019). Ultraselective Antibiotic Sensing with Complementary Strand DNA Assisted Aptamer/MoS_2_ Field-Effect Transistors. Biosens. Bioelectron..

[134] Zheng C., Jin X., Li Y., Mei J., Sun Y., Xiao M., Zhang H., Zhang Z., Zhang G.-J. (2019). Sensitive Molybdenum Disulfide Based Field Effect Transistor Sensor for Real-Time Monitoring of Hydrogen Peroxide. Sci. Rep..

[135] Sakthivel K., Govindasamy M., Chen S., Muthumariappan A., Mani V., Chen T.-W., Selvaraj S. (2017). MWCNTs/MoS_2_ Decorated Cobalt Oxide Polyhedrons Composite Film Modified Electrode for Electrochemical Determination of Dopamine in Rat Brain and Human Blood Serum Samples. Int. J. Electrochem. Sci..

[136] Su S., Cao W., Liu W., Lu Z., Zhu D., Chao J., Weng L., Wang L., Fan C., Wang L. (2017). Dual-Mode Electrochemical Analysis of MicroRNA-21 Using Gold Nanoparticle-Decorated MoS_2_ Nanosheet. Biosens. Bioelectron..

[137] Chand R., Ramalingam S., Neethirajan S. (2018). A 2D Transition-Metal Dichalcogenide MoS_2_ Based Novel Nanocomposite and Nanocarrier for Multiplex MiRNA Detection. Nanoscale.

[138] Sweeney R.W. (1996). Transmission of Paratuberculosis. Vet. Clin. N. Am. Food Anim. Pract..

[139] Benchimol S., Fuks A., Jothy S., Beauchemin N., Shirota K., Stanners C.P. (1989). Carcinoembryonic Antigen, a Human Tumor Marker, Functions as an Intercellular Adhesion Molecule. Cell.

[140] Wang Y., Wang Y., Wu D., Ma H., Zhang Y., Fan D., Pang X., Du B., Wei Q. (2018). Label-Free Electrochemical Immunosensor Based on Flower-like Ag/MoS_2_/RGO Nanocomposites for Ultrasensitive Detection of Carcinoembryonic Antigen. Sens. Actuators B Chem..

[141] Liu L., Wei Y., Jiao S., Zhu S., Liu X. (2019). A Novel Label-Free Strategy for the Ultrasensitive MiRNA-182 Detection Based on MoS_2_/Ti3C2 Nanohybrids. Biosens. Bioelectron..

[142] Jiang S., Zhang H.-W., Lu M.-H., He X.-H., Li Y., Gu H., Liu M.-F., Wang E.-D. (2010). MicroRNA-155 Functions as an OncomiR in Breast Cancer by Targeting the Suppressor of Cytokine Signaling 1 Gene. Cancer Res..

[143] Liu L., Zhu S., Wei Y., Liu X., Jiao S., Yang J. (2019). Ultrasensitive Detection of MiRNA-155 Based on Controlled Fabrication of AuNPs@MoS_2_ Nanostructures by Atomic Layer Deposition. Biosens. Bioelectron..

[144] Rawat B., Mishra K.K., Barman U., Arora L., Pal D., Paily R.P. (2020). Two-Dimensional MoS_2_-Based Electrochemical Biosensor for Highly Selective Detection of Glutathione. IEEE Sens. J..

[145] Da Costa Ferreira S., Chachá S.G.F., Souza F.F., Teixeira A.C., de Carvalho Santana R., Deghaide N.H.S., Rodrigues S., Marano L.A., Mendes-Junior C.T., Ramalho L.N.Z. (2017). The HLA-G 14-Base Pair Deletion Allele and the Deletion/Deletion Genotype Are Associated with Persistent HBe Antigenemia in Chronic Hepatis B Infection. Hum. Immunol..

[146] Wang T., Zhu R., Zhuo J., Zhu Z., Shao Y., Li M. (2014). Direct Detection of DNA below Ppb Level Based on Thionin-Functionalized Layered MoS_2_ Electrochemical Sensors. Anal. Chem..

[147] Yang T., Chen M., Kong Q., Luo X., Jiao K. (2017). Toward DNA Electrochemical Sensing by Free-Standing ZnO Nanosheets Grown on 2D Thin-Layered MoS_2_. Biosens. Bioelectron..

[148] Zhang W., Dai Z., Liu X., Yang J. (2018). High-Performance Electrochemical Sensing of Circulating Tumor DNA in Peripheral Blood Based on Poly-Xanthurenic Acid Functionalized MoS_2_ Nanosheets. Biosens. Bioelectron..

[149] Zhou Y., Li F., Wu H., Chen Y., Yin H., Ai S., Wang J. (2019). Electrochemical Aptasensing Strategy for Kanamycin Detection Based on Target-Triggered Single-Strand DNA Adsorption on MoS_2_ Nanosheets and Enzymatic Signal Amplification. Sens. Actuators B Chem..

[150] Zhang J., Han D., Wang S., Zhang X., Yang R., Ji Y., Yu X. (2019). Electrochemical Detection of Adenine and Guanine Using a Three-Dimensional WS_2_ Nanosheet/Graphite Microfiber Hybrid Electrode. Electrochem. Commun..

[151] Maes M., Galecki P., Chang Y.S., Berk M. (2011). A Review on the Oxidative and Nitrosative Stress (O&NS) Pathways in Major Depression and Their Possible Contribution to the (Neuro)Degenerative Processes in That Illness. Prog. Neuro-Psychopharmacol. Biol. Psychiatry.

[152] Reuter S., Gupta S.C., Chaturvedi M.M., Aggarwal B.B. (2010). Oxidative Stress, Inflammation, and Cancer: How Are They Linked?. Free. Radic. Biol. Med..

[153] Wang T., Zhu H., Zhuo J., Zhu Z., Papakonstantinou P., Lubarsky G., Lin J., Li M. (2013). Biosensor Based on Ultrasmall MoS_2_ Nanoparticles for Electrochemical Detection of H2O2 Released by Cells at the Nanomolar Level. Anal. Chem..

[154] Ma D., Yu J., Yin W., Zhang X., Mei L., Zu Y., An L., Gu Z. (2018). Synthesis of Surface-Modification-Oriented Nanosized Molybdenum Disulfide with High Peroxidase-Like Catalytic Activity for H2O2 and Cholesterol Detection. Chem.—A Eur. J..

[155] Shu Y., Zhang W., Cai H., Yang Y., Yu X., Gao Q. (2019). Expanding the Interlayers of Molybdenum Disulfide toward the Highly Sensitive Sensing of Hydrogen Peroxide. Nanoscale.

[156] Wood A., O’Neal D., Furler J., Ekinci E.I. (2018). Continuous Glucose Monitoring: A Review of the Evidence, Opportunities for Future Use and Ongoing Challenges. Intern. Med. J..

[157] Pu Z., Zou C., Wang R., Lai X., Yu H., Xu K., Li D. (2016). A Continuous Glucose Monitoring Device by Graphene Modified Electrochemical Sensor in Microfluidic System. Biomicrofluidics.

[158] Facchinetti A., Sparacino G., Guerra S., Luijf Y.M., DeVries J.H., Mader J.K., Ellmerer M., Benesch C., Heinemann L., Bruttomesso D. (2013). Real-Time Improvement of Continuous Glucose Monitoring Accuracy: The Smart Sensor Concept. Diabetes Care.

[159] Lee H., Hong Y.J., Baik S., Hyeon T., Kim D.-H. (2018). Enzyme-Based Glucose Sensor: From Invasive to Wearable Device. Adv. Healthc. Mater..

[160] Huang K.-J., Liu Y.-J., Liu Y.-M., Wang L.-L. (2014). Molybdenum Disulfide Nanoflower-Chitosan-Au Nanoparticles Composites Based Electrochemical Sensing Platform for Bisphenol A Determination. J. Hazard. Mater..

[161] Su S., Lu Z., Li J., Hao Q., Liu W., Zhu C., Shen X., Shi J., Wang L. (2018). MoS_2_–Au@Pt Nanohybrids as a Sensing Platform for Electrochemical Nonenzymatic Glucose Detection. New J. Chem..

[162] Kong R.-M., Ding L., Wang Z., You J., Qu F. (2015). A Novel Aptamer-Functionalized MoS_2_ Nanosheet Fluorescent Biosensor for Sensitive Detection of Prostate Specific Antigen. Anal. Bioanal. Chem..

[163] Dhenadhayalan N., Yadav K., Irulappan Sriram M., Lee H.-L., Lin K.-C. (2017). Ultra-Sensitive DNA Sensing of a Prostate-Specific Antigen Based on 2D Nanosheets in Live Cells. Nanoscale.

[164] Xi Q., Zhou D.-M., Kan Y.-Y., Ge J., Wu Z.-K., Yu R.-Q., Jiang J.-H. (2014). Highly Sensitive and Selective Strategy for MicroRNA Detection Based on WS_2_ Nanosheet Mediated Fluorescence Quenching and Duplex-Specific Nuclease Signal Amplification. Anal. Chem..

[165] Cai B., Guo S., Li Y. (2018). MoS_2_-Based Sensor for the Detection of MiRNA in Serum Samples Related to Breast Cancer. Anal. Methods.

[166] Catalán-Gómez S., Briones M., Cortijo-Campos S., García-Mendiola T., de Andrés A., Garg S., Kung P., Lorenzo E., Pau J.L., Redondo-Cubero A. (2020). Breast Cancer Biomarker Detection through the Photoluminescence of Epitaxial Monolayer MoS_2_ Flakes. Sci. Rep..

[167] Takada M., Masuda N., Matsuura E., Kusunoki Y., Matui K., Nakagawa K., Yana T., Tuyuguchi I., Oohata I., Fukuoka M. (1995). Measurement of Cytokeratin 19 Fragments as a Marker of Lung Cancer by CYFRA 21-1 Enzyme Immunoassay. Br. J. Cancer.

[168] Zhao L., Cheng M., Liu G., Lu H., Gao Y., Yan X., Liu F., Sun P., Lu G. (2018). A Fluorescent Biosensor Based on Molybdenum Disulfide Nanosheets and Protein Aptamer for Sensitive Detection of Carcinoembryonic Antigen. Sens. Actuators B Chem..

[169] Kenry, Geldert A., Zhang X., Zhang H., Lim C.T. (2016). Highly Sensitive and Selective Aptamer-Based Fluorescence Detection of a Malarial Biomarker Using Single-Layer MoS_2_ Nanosheets. ACS Sens..

[170] Zhang Y., Zheng B., Zhu C., Zhang X., Tan C., Li H., Chen B., Yang J., Chen J., Huang Y. (2015). Single-Layer Transition Metal Dichalcogenide Nanosheet-Based Nanosensors for Rapid, Sensitive, and Multiplexed Detection of DNA. Adv. Mater..

[171] Rahman M.S., Hasan M.R., Rikta K.A., Anower M.S. (2018). A Novel Graphene Coated Surface Plasmon Resonance Biosensor with Tungsten Disulfide (WS_2_) for Sensing DNA Hybridization. Opt. Mater..

[172] Ge J., Ou E.-C., Yu R.-Q., Chu X. (2014). A Novel Aptameric Nanobiosensor Based on the Self-Assembled DNA–MoS_2_ Nanosheet Architecture for Biomolecule Detection. J. Mater. Chem. B.

[173] Loan P.T.K., Zhang W., Lin C.-T., Wei K.-H., Li L.-J., Chen C.-H. (2014). Graphene/MoS_2_ Heterostructures for Ultrasensitive Detection of DNA Hybridisation. Adv. Mater..

[174] Huang Y., Shi Y., Ying Yang H., Ai Y. (2015). A Novel Single-Layered MoS_2_ Nanosheet Based Microfluidic Biosensor for Ultrasensitive Detection of DNA. Nanoscale.

[175] Wang S., Zhang Y., Ning Y., Zhang G.-J. (2015). A WS 2 Nanosheet-Based Platform for Fluorescent DNA Detection via PNA–DNA Hybridization. Analyst.

[176] Xu B., Su Y., Li L., Liu R., Lv Y. (2017). Thiol-Functionalized Single-Layered MoS_2_ Nanosheet as a Photoluminescence Sensing Platform via Charge Transfer for Dopamine Detection. Sens. Actuators B Chem..

[177] Gao L., Li Q., Deng Z., Brady B., Xia N., Zhou Y., Shi H. (2017). Highly Sensitive Protein Detection via Covalently Linked Aptamer to MoS_2_ and Exonuclease-Assisted Amplification Strategy. Int. J. Nanomed..

[178] Hosseini S., Vázquez-Villegas P., Rito-Palomares M., Martinez-Chapa S.O., Hosseini S., Vázquez-Villegas P., Rito-Palomares M., Martinez-Chapa S.O. (2018). Advantages, Disadvantages and Modifications of Conventional ELISA. Enzyme-Linked Immunosorbent Assay (ELISA): From A to Z.

[179] Bleicher A.V., Unger H.W., Rogerson S.J., Aitken E.H. (2018). A Sandwich Enzyme-Linked Immunosorbent Assay for the Quantitation of Human Plasma Ferritin. MethodsX.

